# Multi-criteria decision analysis for integrated water quality assessment and management support

**DOI:** 10.1016/j.wroa.2018.100010

**Published:** 2018-11-14

**Authors:** Nele Schuwirth, Mark Honti, Ivana Logar, Christian Stamm

**Affiliations:** a Eawag: Swiss Federal Institute of Aquatic Science and Technology, Überlandstrasse 133, 8600, Dübendorf, Switzerland; b MTA-BME Water Research Group, Hungarian Academy of Sciences, Műegyetem rkp 3, 1111, Budapest, Hungary

**Keywords:** Integrated assessment, Uncertainty, Water quality modelling, Scenario analysis, Cost-effectiveness analysis, Micropollutants

## Abstract

In densely populated areas, surface waters are affected by many sources of pollution. Besides classical pollutants like nutrients and organic matter that lead to eutrophication, micropollutants from various point- and non-point sources are getting more attention by water quality managers. For cost-effective management an integrated assessment is needed that takes into account all relevant pollutants and all sources of pollution within a catchment. Due to the difficulty of identifying and quantifying sources of pollution and the need for considering long-term changes in boundary conditions, typically substantial uncertainty exists about the consequences of potential management alternatives to improve surface water quality. We therefore need integrated assessment methods that are able to deal with multiple objectives and account for various sources of uncertainty.

This paper aims to contribute to integrated, prospective water management by combining a) multi-criteria decision support methods to structure the decision process and quantify preferences, b) integrated water quality modelling to predict consequences of management alternatives accounting for uncertainty, and c) scenario planning to consider uncertainty from potential future climate and socio-economic developments, to evaluate the future cost-effectiveness of water quality management alternatives at the catchment scale. It aims to demonstrate the usefulness of multi-attribute value functions for water quality assessment to i) propagate uncertainties throughout the entire assessment procedure, ii) facilitate the aggregation of multiple objectives while avoiding discretization errors when using categories for sub-objectives, iii) transparently communicate the results. We show how to use such multi-attribute value functions for model-based decision support in water quality management.

We showcase the procedure for the Mönchaltorfer Aa catchment on the Swiss Plateau. We evaluate ten different water quality management alternatives, including current practice, that tackle macro- and micropollutants from a wide spectrum of agricultural and urban sources. We evaluate costs and water quality effects of the alternatives under four different socio-economic scenarios for the horizon 2050 under present and future climate projections and visualize their uncertainty. While the performance of alternatives is catchment specific, the methods can be transferred to other places and other management situations. Results confirm the need for cross-sectoral coordination of different management actions and interdisciplinary collaboration to support the development of prospective strategies to improve water quality.

## Highlights

• Model based decision support is needed for integrative water quality management.
• Multi-criteria decision support methods help developing water quality assessments.
• We evaluate alternatives that reduce agricultural and urban sources of pollution.
• We illustrate tradeoffs between costs and water quality including uncertainty.
• Scenario analysis helps evaluating robustness of outcomes to future developments.

## Graphical abstract

**Figure:**
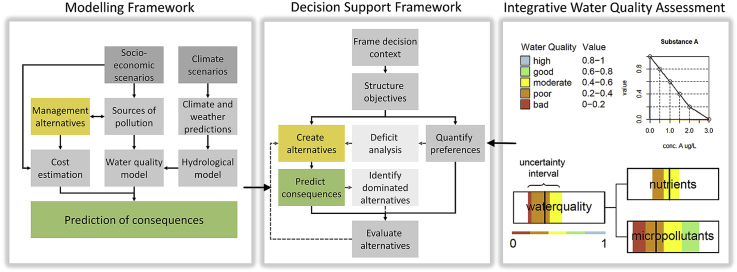

## 1 Introduction

Water quality issues in surface waters are caused by many different point- and non-point sources from urban and agricultural areas (Howarth et al., 2005; Conley et al., 2009; Wittmer et al., 2010). Pollution of surface waters is caused by various substances, including nutrients, heavy metals, and organic micropollutants (Ippolito et al., 2015; Destouni et al., 2017; Munz et al., 2017; Spycher et al., 2018). As surface waters can only be efficiently managed on the catchment scale and in an integrative manner (Agarwal et al., 2000), all polluting sources and substances should be considered to make (cost-)effective decisions in water quality management. Such decisions require a catchment scale perspective and need to account for future changes due to factors that are out of scope of water quality management, like climate change and socio-economic development in the catchment. Ideally, management alternatives would be chosen according to their contribution for improving the overall water quality in the catchment, their costs and perhaps other relevant societal objectives.

Multi-criteria decision analysis (MCDA) offers numerous methods to support structured decision making and to combine multiple criteria to an overall assessment (Belton and Stewart, 2002; Gregory et al., 2012). These methods are increasingly used to support environmental management decisions (Kiker et al., 2005). The aims of such applications are manifold and include a) facilitating participation and a mutual learning process, b) identifying pros and cons of different management alternatives, c) identification of consensus solutions that fulfill the management objectives as good as possible. Major challenges in applying such methods in real world cases include the prediction of consequences of management alternatives regarding their fulfillment of objectives and the quantification of preferences of decision makers, stakeholders or the public regarding the different objectives (Reichert et al., 2015).

In the field of water quality management, a current review documents large variability in the effectiveness of different management alternatives (Liu et al., 2017) that are largely context dependent. To quantify the current state of knowledge about their expected effectiveness, integrated water quality models are needed (Honti et al., 2017). Because such predictions may yield high uncertainty, the decision support framework needs to account for these uncertainties and provide guidance how to deal with them to support rational decision making in environmental management (Wardekker et al., 2008; Sigel et al., 2010; Warmink et al., 2010). Uncertainty assessment was identified as one of the key issues for integrated assessment and modelling (Hamilton et al., 2015) and several typologies for the description of different aspects of uncertainty assessment have been developed (e.g.Walker et al., 2003; Warmink et al., 2010).

In this paper, we introduce a model-based decision support framework for water quality management that combines the assessment of water quality management alternatives with uncertain consequences regarding multiple objectives with scenario planning to account for impacts of future developments that are out of scope of water quality management. We therefore explicitly deal here with two major sources of uncertainty, the future development of external boundary conditions (climate and socio-economic development) and uncertainty of model predictions about the consequences of management alternatives conditional on the boundary conditions (Honti et al., 2017).

We use multi-attribute value theory (Dyer and Sarin, 1979; Eisenfuehr et al., 2010) to formulate a value function that describes the preferences regarding the fulfillment of the objectives including trade-offs between objectives. Unlike in standard examples for decision support, the preferences regarding water quality do not reflect subjective preferences of decision makers or stakeholders, but should reflect the current state of knowledge regarding the effects of different substances on ecosystem and human health. They have to account for legal provisions and will be based on existing water quality assessment procedures. These assessment procedures are based on “immission standards”, i.e. they assess the water quality in the receiving water and therefore allow integrated assessment of different sources of impairment. In contrast, “emission standards” assess each point source separately (Freni et al., 2010), which is helpful for source control, but does not consider the combined effects of different sources of pollution.

We illustrate the framework with an application to a catchment on the densely populated Swiss Plateau that is affected by urban and agricultural point and non-point source pollution (Honti et al., 2017). In this case study we aim for evaluating costs and effects of multiple water management alternatives to reduce the input of nutrients and micropollutants in surface waters under four different scenarios of socio-economic development under current and future climate conditions with a time horizon of 2050. The ten management alternatives considered include a business-as-usual alternative and a combination of measures that tackles all sources of pollution.

While the outcomes of this case study will be very case specific and context dependent, the decision support framework and assessment methods accounting for uncertainties of model predictions and of changing boundary conditions are largely independent of the application case and therefore transferable to other cases. With this study we aim for demonstrating how to facilitate a cross-sectoral coordination of long-term water quality management in face of large uncertainties with the help of multi-criteria decision theory.

## 2 Methods

### 2.1 Multi-criteria decision analysis concept

**Fig. 1:**
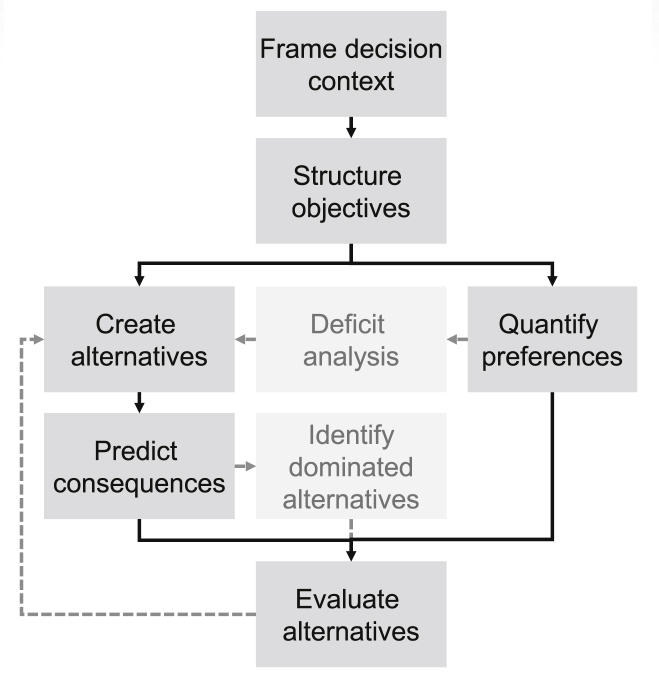
General decision support framework, adapted from Schuwirth et al. (2012) and Reichert et al. (2015).

The general decision support framework we use here consists of 8 steps (Fig. 1) and is based on the concept of *value focused thinking* (Keeney, 1996). In this section we briefly outline these steps, which will be illustrated in more detail by means of a concrete application below.

The first step consists of a definition of the decision context and the scope of the decision. This includes a clarification of people to be involved in the decision making process and their roles as well as important boundary conditions (e.g. regarding the spatial and temporal scale). In the next step, the fundamental objectives are identified that should be fulfilled by the management alternatives. These should include all important decision criteria to be considered. The objectives are structured in form of a hierarchy to facilitate the discussion among stakeholders and the quantification of preferences.

The next steps can be carried out in different orders or even in parallel. They consist of the selection of management alternatives that should be assessed and on the quantification of preferences, in this case the definition of a so called *measurable value function* (Dyer and Sarin, 1979) that describes the degree of fulfillment of objectives based on measurable attributes. Such a value function maps the attribute levels to an interval scale between 0 and 1, which reflects 0%–100% fulfillment of the objective. A value of zero corresponds to the worst case level of the attribute and 1 to the best level that the attribute can take (see Fig. S1 for examples). In general, apart from these two anchor points, the value function can take any form that represents the preferences of the decision maker and is not restricted to any formal assumption (e.g. linearity or monotonicity). If different stakeholders should be involved in the decision support process, we can elicit a value function for each person or stakeholder group. Applying the value function(s) to the current state allows identifying current deficits regarding the fulfilment of objectives. The identification of sub-objectives that are currently not fulfilled can inspire the creation of management alternatives (Reichert et al., 2015), especially at the lowest level of the objectives hierarchy, where the objectives are very concrete. For example, the identification of specific substances that currently exceed the legal thresholds, may help identifying potential management alternatives, if they can be linked to specific sources of pollution. In some cases, the quantification of preferences may be easier, if the prediction of consequences is already available. In this case, sensitivity analyses can help to focus the elicitation of preferences on the most relevant parts of the value function (Scholten et al., 2015).

The prediction of consequences of any given alternative should be based on the current state of knowledge and be done as objective as possible, whereas the quantification of preferences usually reflects the subjective values of the decision maker or stakeholders. If the fulfillment of the objectives monotonically increases or decreases with the measurable attributes (i.e. the more the better or the less the better), a dominance analysis can reveal, if there are any alternatives that are equal or inferior to others in all aspects and inferior in at least one aspect, even without knowing the full preference structure (Eisenfuehr et al., 2010). However, conflicting objectives are typical for environmental decision making. For example, alternatives might be either more effective or less costly. In this case, the trade-off that people are willing to make between costs and effects, decides, which alternative is preferred: the cheaper or the more effective one. In the last step, all alternatives are evaluated based on the prediction of consequences and the quantified preferences of all stakeholders. This step reveals how well the different alternatives fulfill the objectives and if there are consensus-solutions that satisfy all stakeholders or strong conflicts. The analysis of deficits of certain alternatives may also stimulate the creation of new alternatives, which would lead to an iterative procedure (Hostmann et al. 2005a, 2005b).

### 2.2 Water quality modelling concept

**Fig. 2:**
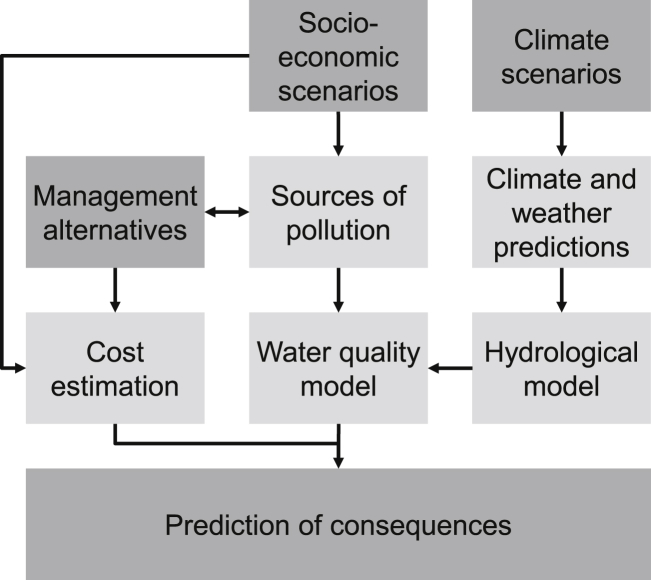
Water quality modelling concept to predict consequences regarding water quality and costs of management alternatives under future climate and socio-economic scenarios.

To predict future consequences of management alternatives regarding costs and water quality, we need to account also for future changes in boundary conditions that are out of scope of local water quality management. These include climate change that will affect hydrological conditions as well as socio-economic changes that may affect management costs and sources of pollution. Since it is difficult to predict the local effects of both climate change and socio-economic changes for a time-horizon of several decades, we use scenarios that try to cover different realizations of the future without specifying how probable they are (Lienert et al., 2015). We use these scenarios as inputs into hydrological and water quality models as well as for cost estimates quantifying conditional probabilities for cost and effects conditional on these scenarios (Fig. 2). According to the terminology of Warmink et al. (2010), the *locations of uncertainty*, which are quantified by the water quality model, cover input, model structure, and parameter uncertainty (Honti et al., 2017). The *levels of uncertainty* can be classified into scenario uncertainty regarding external (socio-economic and climate) conditions and statistical uncertainty regarding the water quality model outcomes and costs. The *nature of uncertainty* is dominated by epistemic uncertainty (imperfect knowledge) but also includes natural variability of the system (aleatory uncertainty).

### 2.3 Introduction to case study

**Fig. 3:**
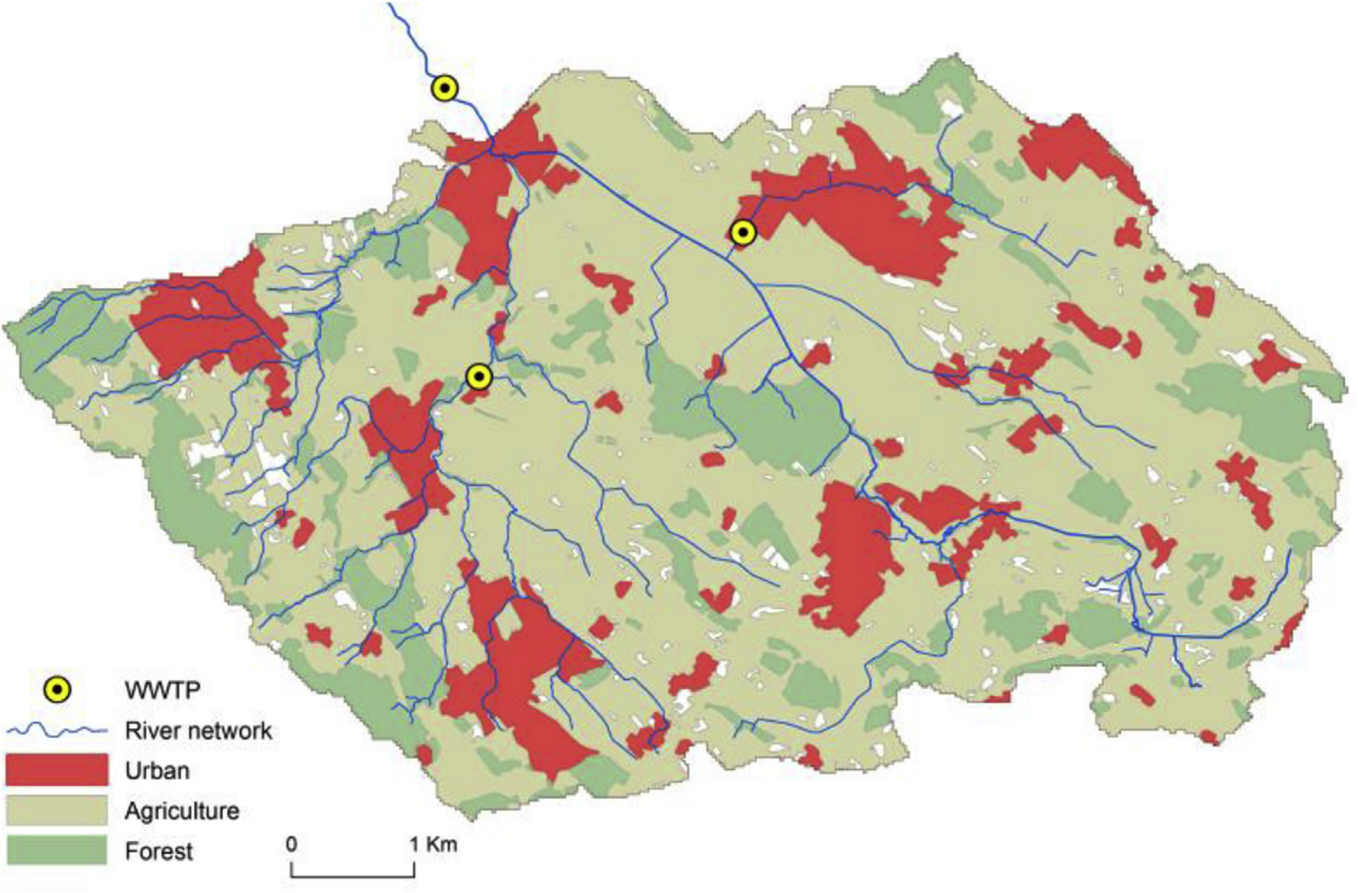
Land use and location of wastewater treatment plants (WWTP) in the Mönchaltorfer Aa catchment.

We concretize these concepts with a case study in the Mönchaltorfer Aa catchment at the densely populated Swiss Plateau. The goal of this case study is to assess the performance of different water quality management strategies to improve water quality at the outlet of the Mönchaltorfer Aa catchment with a time horizon of 2050. The catchment has an extent of 43 km^2^ with currently 24000 inhabitants living in five municipalities. Intensive agriculture (57%), forest (15%) and urban settlements (11%) are most important land uses (GEOSTAT, 1997) (see Fig. 3). The lowest point of the case study area is at 440 m above sea level. The fraction of treated wastewater in the Mönchaltorfer Aa is 30–50% of the mean river discharge of 1.1 m^3^/s. The Mönchaltorfer Aa flows into Greifensee, a eutrophic lake with a surface of 8.5 km^2^ and an average depth of 18 m (Bundesamt für Umwelt (BAFU) 2016).^1^

### 2.4 Structuring objectives

**Fig. 4:**
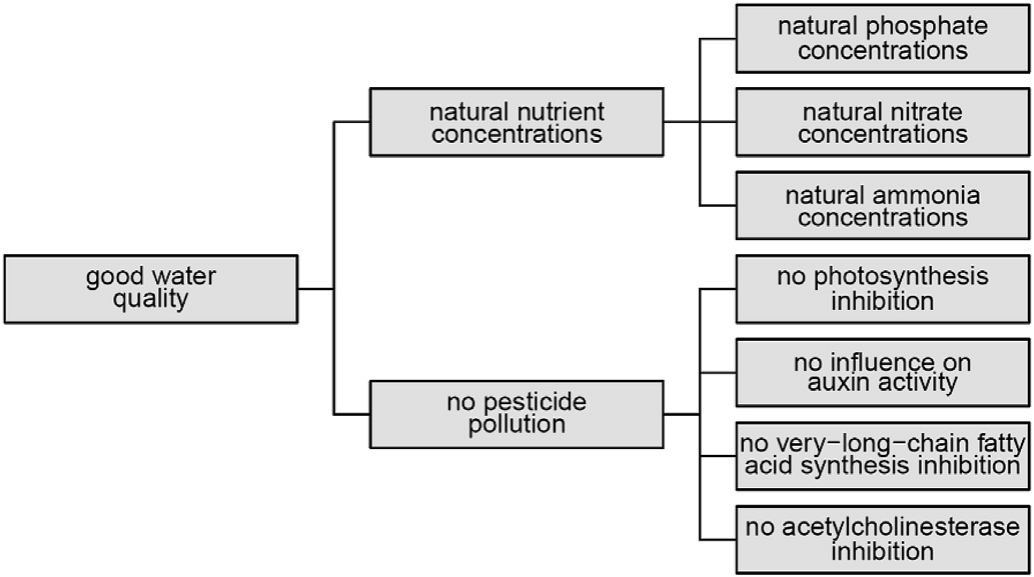
Objectives hierarchy for the water quality assessment regarding nutrients and pesticides, restricted to objectives with available attribute predictions, see text for explanation.

The two fundamental objectives in this decision context are a good water quality and low costs. Sub-objectives regarding water quality include natural nutrient concentrations and no pollution by organic micropollutants and heavy metals. Here we present the objectives hierarchy restricted to objectives regarding nutrients and pesticides for which the attributes could be predicted within this study (Fig. 4) (Honti et al., 2017). To account for mixture toxicity of pesticides, the different pesticides are grouped according to their toxic modes of action (which are photosynthesis inhibition, influence on auxin activity, very-long-chain-fatty-acid synthesis inhibition and acetylcholinesterase inhibition). The objectives are formulated accordingly. An alternative approach suggests to group the micropollutants according to the most sensitive target organism groups, which would be straight forward to implement as well (Junghans et al., 2013).

### 2.5 Quantification of preferences

Preferences regarding different objectives are generally subjective. They may differ between decision makers and stakeholder groups and depend on the context of the decision situation. As described above, we can derive them by eliciting multi-attribute value functions from the stakeholders in face to face interviews (Eisenfuehr et al., 2010) or in combination with online questionnaires (Scholten et al., 2015).

However, in the context of water quality management, it makes sense to use existing water quality assessment procedures that were developed to describe the water quality based on substance concentrations, since it should reflect legislative constraints and potential effects of the different pollutants on the ecosystem. Existing immission oriented water quality assessment methods in Switzerland and the EU are usually based on 5 color-coded water quality classes (European Union, 2000) (Table 1).

**Table 1:**
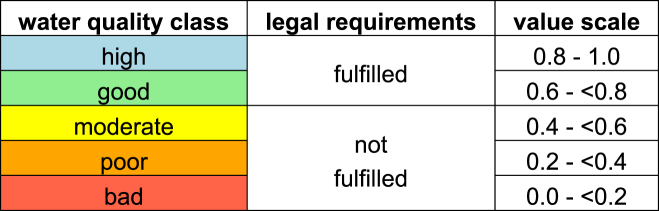
Water quality classes and their translation to the value scale.

We show here that it is useful to translate the existing water quality assessment procedures into so called *measurable value functions* that describe the degree of the fulfillment of the objectives from 0 to 100% (Langhans et al., 2013). This ensures a consistent approach for a continuous evaluation (instead of only discrete classes) that avoids the propagation of discretization errors. Furthermore, it facilitates the propagation of uncertainties through all levels of the objectives hierarchy, the identification of appropriate methods to aggregate the values of sub-objectives to higher-level objectives (Langhans et al., 2014; Haag et al. 2018), and a transparent and consistent communication of the results.

For nutrients, we used a translation of the nutrient module of the Swiss modular concept for stream assessment (Bundi et al., 2000; Liechti, 2010) expressed as a value function (see Fig. S1). The assessment procedure for pesticides is based on a method used by the cantonal authority in charge of water quality management in the case study region (AWEL, 2006). Pesticides are grouped according to their mode of action and compared to their chronic and acute environmental quality standards (EQS) (Table S2) to derive a risk quotient that takes into account the toxic potency of each substance. The establishment of environmental quality standards for pesticides in Switzerland are currently in the political consultation and the development of a national assessment procedure based on biweekly composite samples is currently under development.

The attributes for all water quality parameters range from a concentration of 0 (unpolluted water, corresponding to 1 on the value scale) to the worst case to be expected in Swiss rivers (corresponding to 0 on the value scale). The legal thresholds, as defined by the Swiss water protection legislation, correspond to a value of 0.6 on the value scale (Table 1).

**Fig. 5:**
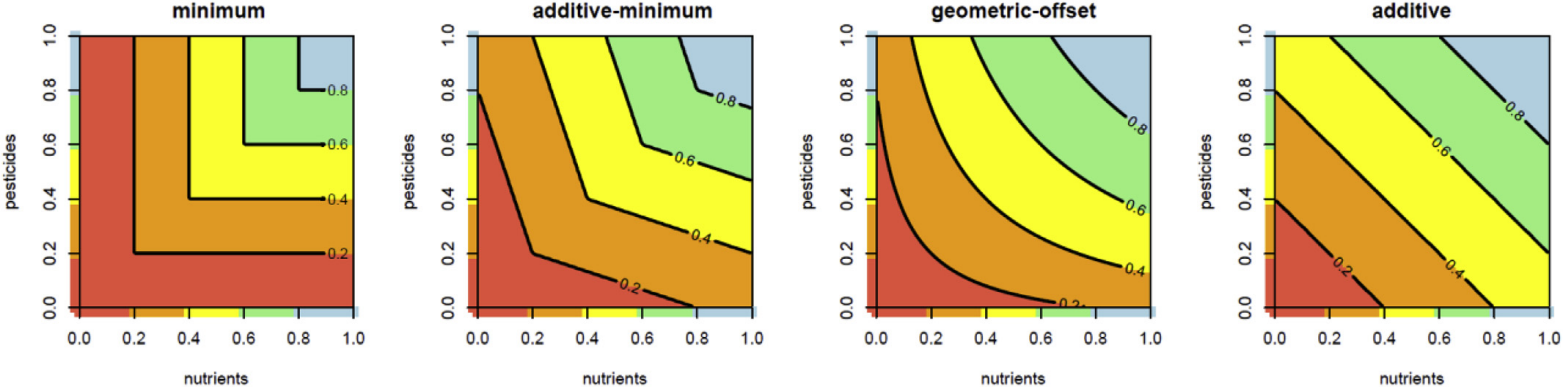
Different aggregation methods for the overall water quality assessment based on nutrients and pesticides using equal weights. Contour lines show the aggregated value and colors the water quality classes from poor (red) to high (blue). Additive-minimum aggregation with parameter α=0.5 (second panel) and geometric-offset aggregation with parameter δ=0.1 (third panel). (For interpretation of the references to color in this figure legend, the reader is referred to the Web version of this article.)

On the higher levels of the objectives hierarchy (Fig. 4), we have to find aggregation functions that describe, how the fulfillment of each higher-level objective depends on its sub-objectives. For ecological assessments, the most often used aggregation functions are the additive aggregation (i.e. weighted arithmetic mean) and minimum aggregation (also called worst case or one out – all out) (Table 2, Fig. 5). While additive aggregation allows for full compensation between good and bad sub-objectives, the minimum aggregation reflects the value of the worst sub-objective only and is insensitive to improvements or deteriorations, if they do not affect the worst sub-objective. Since neither properties are satisfactory in this context (Langhans et al., 2014; Haag et al. 2018), we propose here two other aggregation functions that are a compromise between these extremes: the additive-minimum and the geometric-offset aggregation (Table 2, Fig. 5). Both aggregation functions allow only partial compensation between good and bad sub-objectives but are able to reflect changes in all sub-objectives, according to their weights and parameters (Haag et al. 2018).

**Table 2:** Functions for aggregating the values of sub-objectives to the higher-level objective.

minimum aggregation	$v_{min}=\min\left(v\right)\;$ with $v=\left(v_{1},\cdot \cdot \cdot ,\;v_{n}\right)$ values of the sub-objectives to be aggregated
additive aggregation	weighted arithmetic mean $v_{add}=\;\sum_{i=1}^{n}w_{i}v_{i}$ with weights, $w_{i}\text{,}$ of the sub-objectives i summing up to 1

additive-minimum aggregation	$v_{add-\min}=\;\alpha \cdot v_{add}+\left(1-\alpha \right)\cdot v_{\min}$ with α parameter between 0 and 1 that determines the contribution of the additive aggregation to the overall value

geometric-offset aggregation	$v_{geo-off}=\left(\prod_{i=1}^{n}{\left(v_{i}+\delta \right)}^{w_{i}}\right)-\delta$ with δ parameter between 0 and infinity that determines how much compensation between sub-objectives is possible: a value of zero leads to the weighted geometric mean (which has the often undesirable property that the aggregated value is 0 as soon as one of the values of the sub-objectives is 0) and a value of infinity leads to the weighted arithmetic mean that allows full compensation between sub-objectives, with weights, $w_{i}\text{,}$ of the sub-objectives i summing up to 1

Most pronounced differences between the aggregation functions exist when one of the sub-objectives is very good and the other is very bad. For the examples given in Fig. 5 (assuming equal weights of both sub-objectives, α=0.5, and δ=0.1, respectively), for $v_{nutrients}=0\;\;$ and $v_{pesticides}=1\;\;$ the aggregated values would be $v_{min}=0$, $v_{add}=0.5$, $v_{add-min}=0.25$, and $v_{geo-off}=0.23$.

We considered four possibilities for confronting water quality effects and costs: (1) To elicit a case specific value function for costs and the trade-offs between costs and water quality effects from stakeholders and decision makers (Eisenfuehr et al., 2010; Haag et al. 2018), (2) to optimize water quality under given budget constraints, (3) to minimize costs for a certain water quality target, and (4) to visualize costs and water quality in trade-off diagrams.

Under the current policy system in Switzerland it is unrealistic to expect that water quality managers will have a combined budget from which they can choose the most suitable management alternative in each catchment. Usually, for each type of management alternative (e.g. addressing agriculture or urban wastewater) there is a separate (often national) policy, which defines targets, budget constraints and financing instruments. However, even in this situation an integrated water quality assessment is needed, because these sectoral policies still usually require a spatial prioritization of measures, which profits from cross-sectoral coordination, and because the managers should be informed about the effects they can expect, which requires an overview about changes regarding all sources of pollution.

We therefore consider the fourth option the most interesting for our case study. However, depending on the policy process, the other three options might be suitable as well and would be straightforward to implement.

The value function was implemented in R (R Development Core Team, 2017), using the packages *utility* (Reichert et al., 2013) and *ecoval* (Schuwirth and Reichert, 2014).

### 2.6 Management alternatives

The management alternatives were chosen to tackle all important sources of pollution by at least one alternative in the catchment (Table 3). Point sources include wastewater treatment plants and combined or separated storm water sewers. Non-point sources include losses from agricultural areas via run-off, spray drift, or drainage systems. The management alternatives include a current practice (or business-as-usual) alternative, as well as a combination of several measures to tackle all known sources of pollution at once. A more detailed description can be found in Honti et al. (2017).

**Table 3:** Management alternatives considered in the case study (Honti et al., 2017).

Category	Name	Description	Rationale
Current practice	CurrPrac	Current practice (or business-as-usual)	(included for comparison)
Material Protection	BanBioc	Banning application of biocides on façades	Reduce biocide loads
Urban Water Infrastructure	StoreVol	Increasing storage volumes in urban drainage systems	Reduce stormwater emissions
	PermPav	Increasing proportion of permeable pavements	Reduce urban runoff
	RetRain	Retention of rainwater from roofs	Reduce urban runoff
End of Pipe	WWTP	Enhancing WWTP treatment efficiency with fourth treatment step	Reduce point source loads of micropollutants
Agriculture	OrgFarm	Exclusively organic farming	Eliminate agricultural pesticides
	NatPark	Conversion of agricultural land into a nature park	Eliminate intensive agriculture and pesticides
	BufZone	Reconstruction of riparian buffer zones	Less erosion, more shading
Total Management	All	Measures addressing all sources combined: (BanBioc, StoreVol, PermPav, RainRet, WWTP, OrgFarm, BufZone)	Best available management

### 2.7 Socio-economic scenarios

We adapted four socio-economic scenarios, which were developed for the time horizon of 2050 in a workshop with stakeholders from four municipalities in the catchment of the Mönchaltorfer Aa (Lienert et al., 2015) and extrapolated them to the whole catchment (Table 4). The scenarios are defined in terms of a change in the mean taxable income of the inhabitants in the catchment based on assumptions about the future economic growth in Switzerland, a change in population, and urban area within the catchment (Lienert et al., 2015). The Status quo scenario serves as a baseline while the Exploding growth and Decline scenarios cover rather extreme changes, which the stakeholders considered possible.

**Table 4:** Socio-economic scenarios for 2050 with estimates of population and urban area from Honti et al. (2017).

	Change in mean income	population	urban area
	%/year	% of today	% of today
Status quo	+0.4	100	100
Moderate growth	+2	+20	+5
Exploding growth	+4	+730	+300
Decline	−1.5	−20	100

### 2.8 Climate scenario

We took the A1B emission scenario from the IPCC 4th assessment report. Considering more emission scenarios did not make sense as the time horizon of predictions lies in the range where cross-scenario differences are still negligible. Ten global and regional climate model (GCM-RCM) couplings were used from the ENSEMBLES data archive to train weather generators for the study site (Honti et al., 2017). The divergence of predictions of the 10 GCM-RCM chains represents climatic uncertainty for the future, which was propagated through the model as described below.

### 2.9 Prediction of consequences

#### 2.9.1 Water quality predictions

A detailed description of the water quality model used for predicting the effects of management alternatives under future scenarios is given in Honti et al. (2017). In short, the model covers traditional water quality parameters and a wide set of organic micropollutants. Out of the physical and water quality parameters handled by the model, only nutrients and certain pesticides are considered here. The model provides a parsimonious, conceptual description of all major pollutant pathways in the urban and agricultural environment, including point and non-point sources and a simplified representation of the urban water infrastructure. Simplicity was an intentional development objective to allow for a full propagation of input, observation and model uncertainty through all calculations and to make scenario development feasible. The model was calibrated and run for the same catchment, the Mönchaltorfer Aa. Model performance during calibration was excellent for traditional pollutants, mediocre for pesticides and just acceptable for biocides (Honti et al., 2017). Prediction uncertainty varied accordingly and is therefore very important to consider. Predictions covered 30 years of continuous daily-step time-series, out of which artificial “grab samples” were taken to mimic the current monitoring procedure and apply the water quality assessment. Thus, for all combinations of management alternatives, climate, and socio-economic scenarios the output of the model was a sample for each attribute derived by Monte Carlo Simulations. This sample was propagated to the multi-attribute value function to derive a sample at the value scale that describes the degree of fulfillment of each management objective. From this we derived the median and the 5% and 95% quantiles to visualize the assessment results and their uncertainty.

#### 2.9.2 Cost estimation

For the purpose of estimating the costs of management alternatives under different socio-economic scenarios, it is assumed that specific management alternatives are declared mandatory within the Mönchaltorfer Aa study area. A detailed description of the cost estimation procedure for each management alternative is provided in the Supplementary material. The costs of urban management alternatives are expressed on an annual basis. For each urban management alternative, we use the most reliable existing data or market prices and provide the minimum, maximum and best cost estimate. The minimum and maximum cost estimates can be considered as the lower and upper bounds of the confidence intervals around the best estimate and are used for the uncertainty analysis. The costs of the agricultural management alternatives can occur due to: (i) a decrease in agricultural income and in workload or (ii) an increase in workload, which reduces the productivity of farming. In the former case, the cost of an alternative represents the difference between the contribution margin under the current conditions and the contribution margin under the new conditions. In the latter case, the costs are estimated as the loss in agricultural income earned per working hour for any workload that exceeds that of the reference situation.

## 3 Results and discussion

In the following sections, we present the consequences of the management alternatives on water quality (3.1), costs (3.2) and trade-offs between water quality effects and costs (3.3). In addition we discuss the prerequisites to transfer the approach to other management cases (3.4).

### 3.1 Water quality

#### 3.1.1 Current state, deficit analysis

**Fig. 6:**
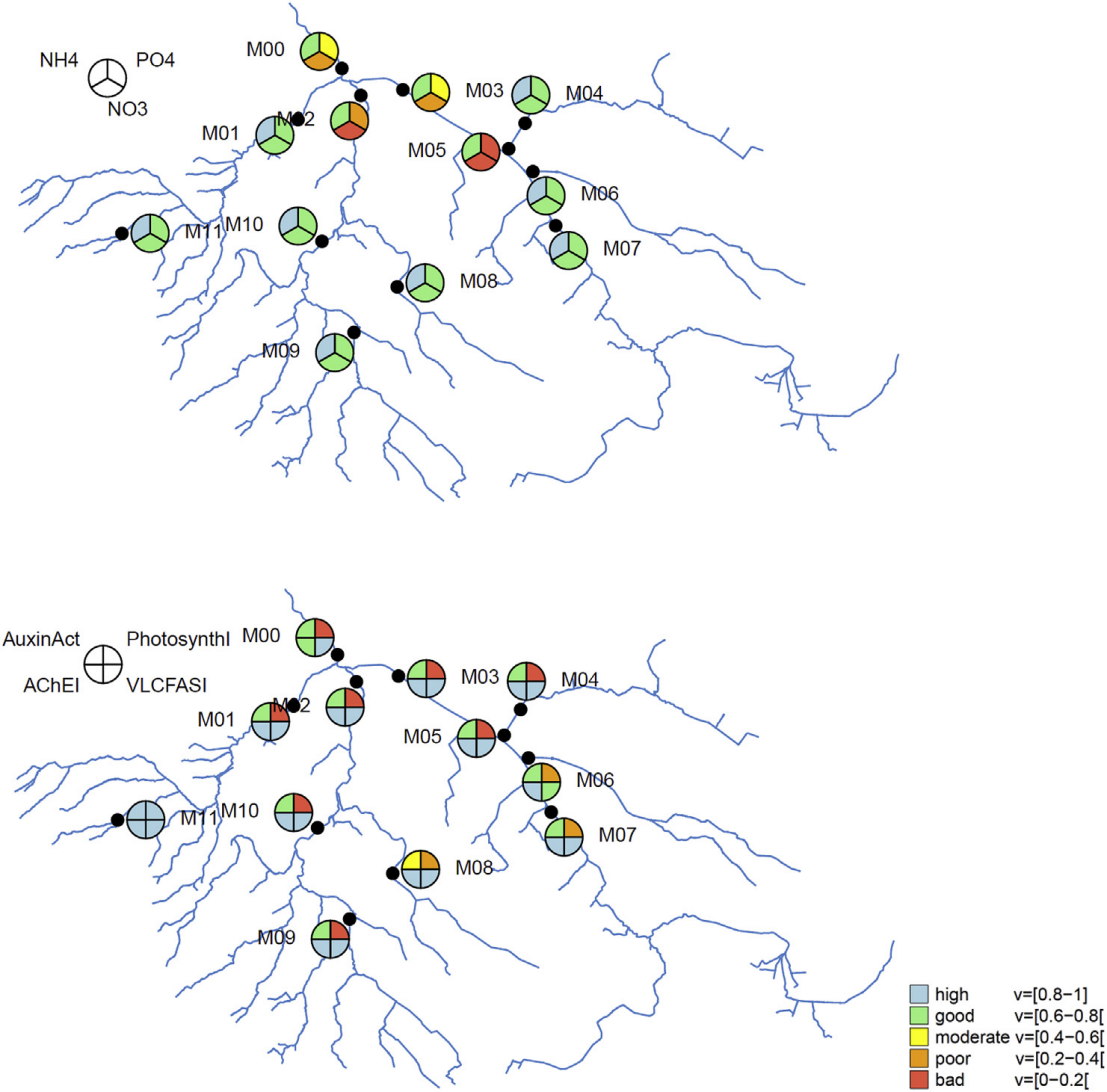
Nutrient (upper panel) and pesticide (lower panel) assessment in the catchment under current practice, present climate and Status quo scenario.

A deficit analysis for the current state reveals that all sites that are influenced by wastewater treatment plants (M00, M02, M03, M05) are affected by nitrate and phosphate pollution (Fig. 6). In addition, most sites in the catchment (all but M11) are impaired by photosynthesis inhibitors.

**Fig. 7:**
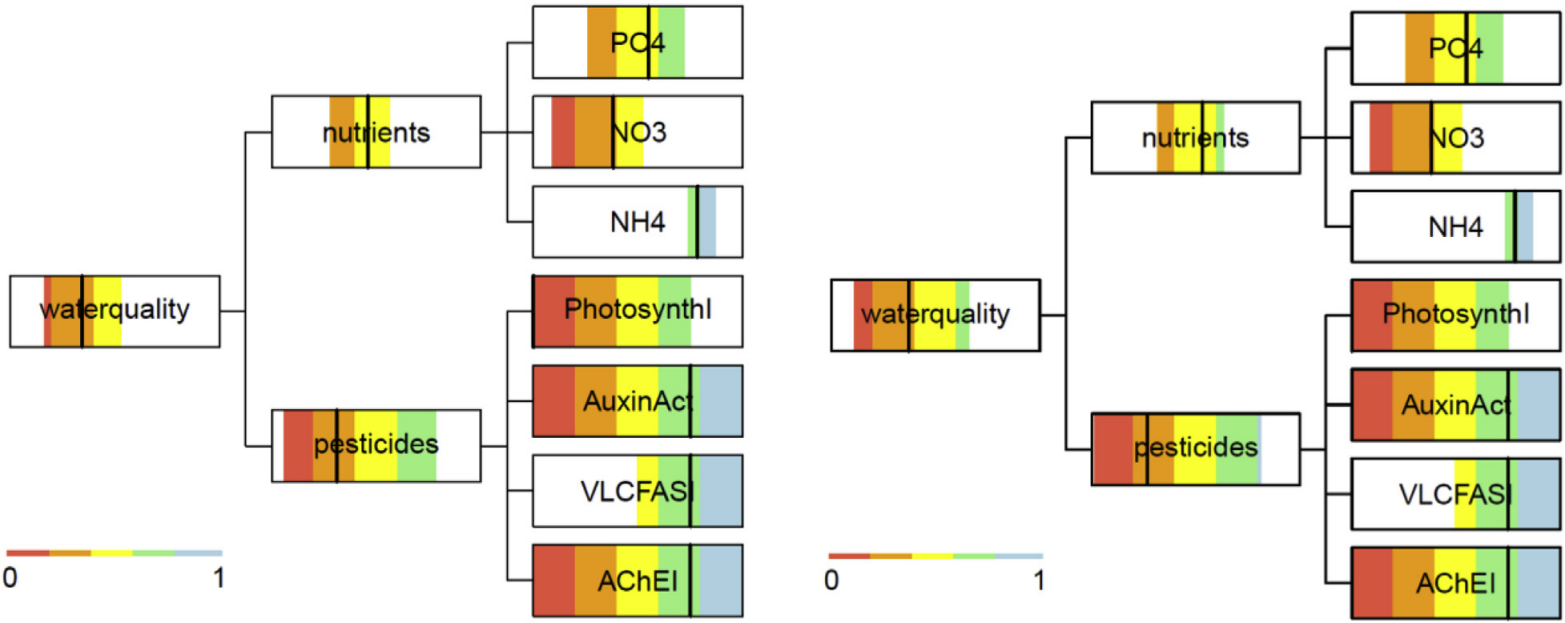
Water quality objectives hierarchy with results for current practice, present climate, Status quo scenario, at catchment outlet (site M00), the vertical black line shows the median and the colored area the 90% uncertainty interval; left panel: additive-minimum aggregation with α = 0.5 for the objectives "good water quality", "low nutrients" and "no pesticides"; right panel: geometric-offset aggregation with δ = 0.1.

The uncertainty assessment for the catchment outlet (Fig. 7) for current practice, present climate, and Status quo scenario reveals that the large uncertainties in pesticides predictions (Honti et al., 2017) lead to large uncertainty regarding the assessment class. Especially for Auxin activity and acetylcholinesterase inhibitors the 90% uncertainty interval covers all five water quality classes from bad to high. The two different aggregation techniques (additive-minimum and geometric-offset) lead to minor differences. In both cases, the overall water quality and pesticide assessment indicates a poor state, the nutrient assessment a moderate state. Since results were similar, in the following we only show the results from additive-minimum aggregation.

#### 3.1.2 Evaluation of alternatives

**Fig. 8:**
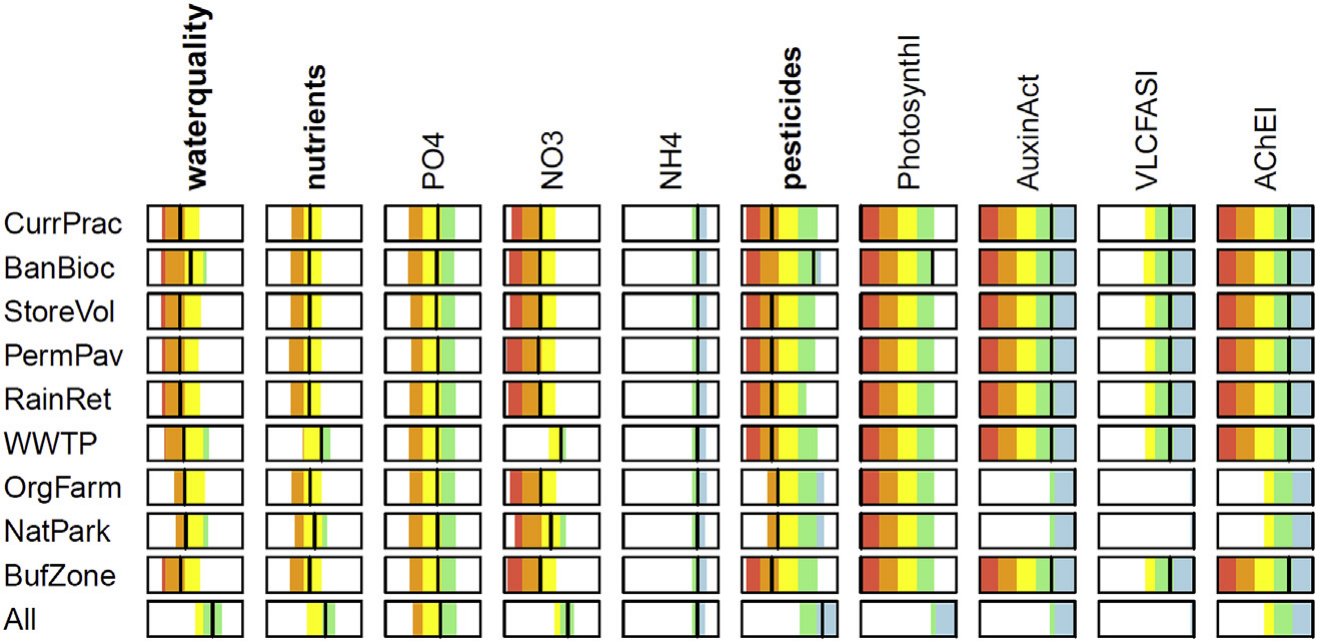
Performance of alternatives at catchment outlet under present climate and Status quo scenario, colored boxes have the same meaning as in Fig. 7 (see legends of Fig. 6, Fig. 7).

We first compare the assessment results for the different management alternatives for the present climate and the Status quo scenario at the catchment outlet (Fig. 8): Only the alternative All that tackles all sources of pollution has a high probability to reach a good water quality. The pesticides sub-objectives AChEI and AuxinAct are already in good to high state for current practice but with large uncertainty. Only the alternatives OrgFarm, NatPark and All are effective in further improving them. The sub-objective VLCFASI is similar but less uncertain. The worst sub-objective of pesticides are the photosynthesis inhibitors PhotosynthI which have a bad mean state but large uncertainty. From the single alternatives only BanBioc can improve it to a mean good state and only All leads to very good state with high certainty. Regarding the aggregated pesticides assessment, BanBioc is the only effective single measure, which leads to a good state but with high uncertainty, and only the combined alterntive All leads to a certain high state. For nutrients, NH_4_ is already good to high under current practice and is unaffected by the management alternatives. The sub-objective PO_4_ has poor to good state under current practice but is unaffected by the management alternatives. NO_3_ is bad to moderate under current practice and significantly improved only by WWTP and All to a good state, while NatPark leads to a minor improvement. Similarly, the overall nutrient assessment has a poor to moderate state for current practice and is significantly improved by WWTP and All to a moderate to good state. The overall water quality assessment is poor to moderate for current practice. The alternatives StoreVol, PermPav, RainRet, BufZone show no effect, the alternatives BanBioc and also WWTP, OrgFarm, and NatPark lead to minor improvements, and only All leads to significant improvement to a good state. Because the respective measures are linked to different policy and management fields (agriculture, urban water management, restrictions on biocide use etc.) this result supports the need for cross-sectorial coordination to achieve a good water quality status. The tools presented here can help communicating expected outcomes including the respective uncertainties in a transparent manner to stakeholders of the different sectors.

**Fig. 9:**
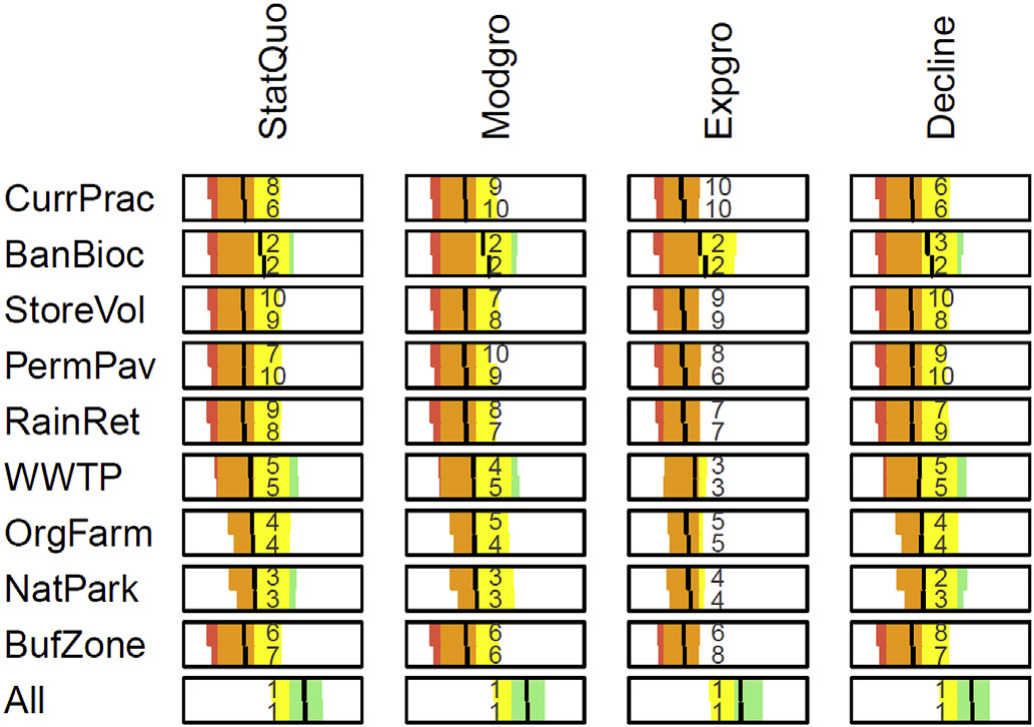
Performance of management alternatives (rows) under socio economic (columns) and climate (future in upper half and present in lower half of each cell) scenarios for the overall water quality assessment at catchment outlet (using additive-minimum aggregation), numbers indicate the ranking of the alternatives in each scenario based on median values.

Next, we compare the influence of climate and socio-economic scenarios on the effects of the management alternatives on the overall water quality assessment (Fig. 9).

Both climate and socio-economic scenarios have rather minor influences on the overall water quality assessment but may affect the ranking of management alternatives based on median values, due to the small differences between some of the alternatives. In general, under future climate the results are expected to be slightly worse than under present climate. While the moderate growth and decline scenarios lead to very similar results compared to the Status Quo, the exponential growth scenario reduces the chance of reaching moderate or good state for all single management alternatives, while the combined alternative All performs similarly to the other socio-economic scenarios. In general, the effectiveness of the management measures appears fairly robust against the potential socio-economic and climate changes considered in this study (Fig. 9).

### 3.2 Costs

**Fig. 10:**
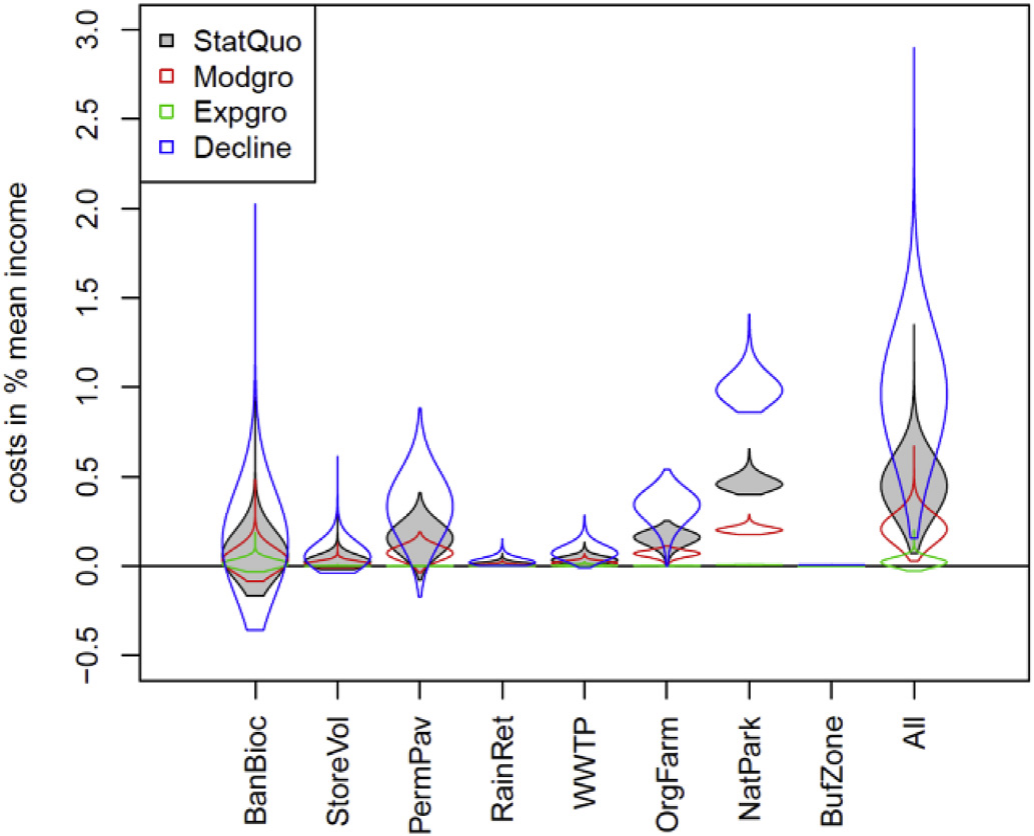
Cost estimates of the different management alternatives expressed as additional costs per inhabitant compared to the current situation in % of the mean taxable income expected under the four different socio-economic scenarios.

The relevance of absolute costs depends on the overall economic situation and the population size, which is reflected in the different socio-economic scenarios. Therefore, the costs presented here (Fig. 10) are expressed per inhabitant and in relative terms, that is, as % of the mean taxable income, which is assumed to differ between socio-economic scenarios (Table 4). Such an approach is expected to better reflect the implications of implementing a management alternative for tax payers under specific socio-economic conditions. The absolute costs are presented in the Supplementary material (Table S16). The influence of the socio-economic scenarios on the costs is much larger than on the water quality. The higher the assumed economic and population growth, the lower the relative costs. The most expensive single alternative under the scenarios StatQuo, Modgro, and Decline is NatPark, due to the loss in agricultural income. Under ExpGro it is BanBioc (but under this scenario all costs are comparably low due to the expected growth of the taxable income at an annual rate of 4%).

### 3.3 Trade-offs between costs and effects

**Fig. 11:**
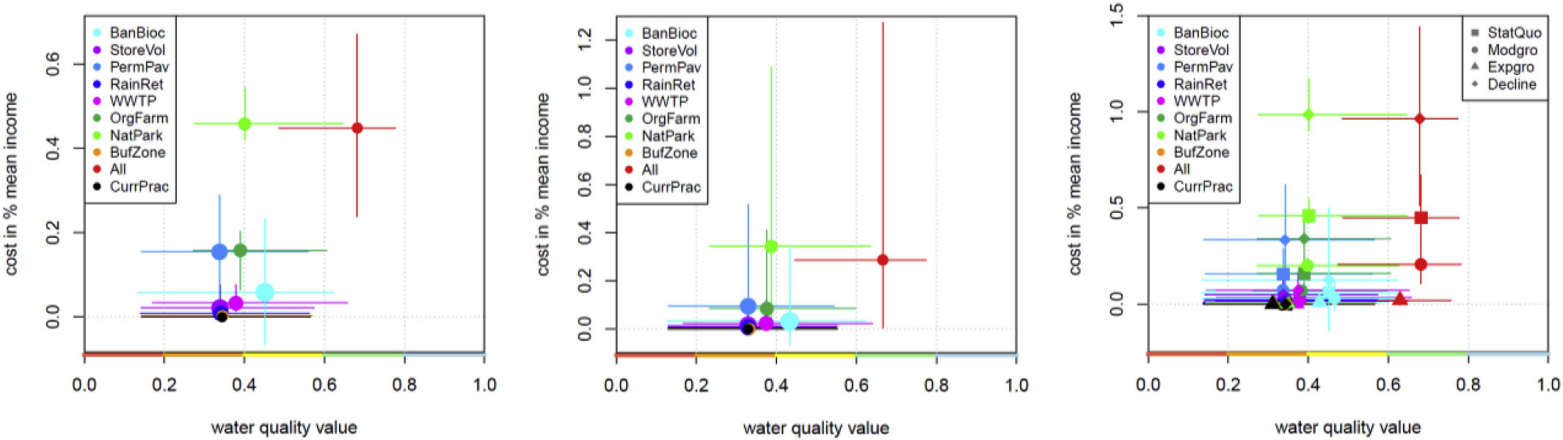
Costs and water quality assessment for different management alternatives under the following scenarios for: (i) present climate and status-quo socio-economic scenario (left panel), (ii) both climate and all four socio-economic scenarios pooled (middle panel), (iii) socio-economic scenarios under the present climate (right panel). Note the different ranges at the y-axis, points show median and lines the 95% uncertainty intervals of cost and water quality estimates.

While the combination of single alternatives that tackle all sources of pollution (All) is the only alternative that can be expected to lead to a good water quality (i.e. median *v* > 0.6) in all future scenarios, it comes with comparably high costs (Fig. 11). Only the nature park alternative (NatPark) comes with similarly high costs, but is much less effective, since it only tackles agricultural sources. While the effects of the management alternatives on water quality can be expected to be rather robust against the future climate and socio-economic scenarios, the costs relative to the mean income vary substantially between socio-economic scenarios (Fig. 11, right panel). From the single alternatives, only the banning of biocides in facades (BanBioc) shows a significant improvement compared to current practice and it comes with comparably low costs.

### 3.4 Transferability of the water quality management framework

From a conceptual point of view, the suggested water quality management framework can be applied to other places and/or other management questions. However, this requires a consideration of each step of the decision support process (Fig. 1) as described in sections 2.1, 2.2 in collaboration with the decision makers, and potentially an adaptation compared to our case study. Since each case may be different, we only briefly discuss here some recommendations for the water quality assessment step. The existing water quality assessment methods differ between countries and we consider here only approaches based on immission standards. Within the EU they usually provide an assessment based on 5 color-coded quality classes according to the Water Framework Directive (WFD Annex V:1.2). In this case, the translation into a continuous function may just require a piecewise linear interpolation between class boundaries, similar to our case study (see Table S1 and Fig. S1), a definition of the concentration that corresponds to a value of 1 (best case, e.g. concentration of 0 for synthetic pollutants or natural background values for geogenic substances), and the worst case concentration that corresponds to a value of 0. In other cases, where only binary pass/fail criteria exist, a continuous value function could be derived based on three anchor points for the concentrations corresponding to a value of 0 and 1 (worst and best case, see above) and the value of 0.6 that corresponds to the legal threshold (if perceived as appropriate). If the development of a 5-class assessment procedure does not seem to be appropriate, only two color-coded classes could be introduced (e.g. red for failing and green for passing the criteria) to visualize the results (see Fig. S6 for an example). However, this approach would then lead to less detailed information. Alternatively, the value functions could be elicited from experts using standard elicitation procedures from multi-criteria decision science (e.g. Eisenfuehr et al., 2010). The hierarchical aggregation could be done as in the presented case study. In any case, if the procedure should inform the decision making process in practice, it requires a consensus about the assessment procedure with decision makers (and stakeholders). If such a consensus cannot be achieved, alternative assessment procedures can be developed (e.g. one for each stakeholder group) and their implications to the results visualized. For practical applications, the objective hierarchy and the value function can be implemented with the R-package *utility* (Reichert et al., 2013), with other decision support software, or just with a simple spreadsheet. An example for the implementation of value functions in R is provided in the Supplementary material (SI section 6).

## 4 Conclusions

Water quality management is a typical example for environmental decision making that has to deal with multiple objectives, many different alternatives, large uncertainties in the prediction of their consequences, and has to account for fairly long-term changes in boundary conditions that cannot directly be influenced by local management, such as socio-economic development or changing climate conditions. For water quality management decisions, it is important to be informed about the changes in substance concentrations that can be expected from different management alternatives. However, since usually many different substances have to be considered that stem from various sources of pollution, an integrated assessment is necessary that helps dealing with the multi-objective nature of the decision problem and helps propagating and visualizing the associated uncertainties. Since the decisions affect many stakeholders, a transparent communication of the decision basis is of particular importance. With this study, we introduced a framework to combine the use of multi-attribute value functions for integrated assessment with water quality modelling and scenario planning. We illustrated its suitability to help informing stakeholders, policy makers, and the public about the decision problem with a full account of the uncertainties of expected outcomes.

A central element of the presented framework is to use a continuous value scale for each sub-objective. This avoids the propagation of discretization errors from the lowest to the highest level of the objectives hierarchy, but still allows translating the values into color-coded quality classes, which have a familiar meaning to the decision makers, at all levels of the objectives hierarchy. This allows choosing the appropriate level of detail for a transparent discussion of the effects of different management alternatives with stakeholders, policy makers, or the public.

While the methods and visualizations used are generalizable to other water quality management decisions, the outcomes of the case study are very catchment specific, because they depend on the local conditions and the contributions of the different sources of pollution, which may vary substantially between catchments. Our results show that, especially when uncertainty at the lowest level of the objectives hierarchy is large and varies between alternatives, it is very insightful to propagate the uncertainty to the value scale and translate it into water quality classes that have a clear meaning for policy makers and managers. The combination with scenario planning for boundary conditions that are hard to predict allows evaluating the robustness of the performance of different management alternatives to future changes.

This study confirms the need for a cross-sectoral coordination between different management actions to achieve larger ecological effects (Smiley et al., 2009; Paillex et al., 2017), even if the funding instruments and implementation strategies depend on sectoral policies.

## Declarations of interest

None.

## Appendix A Supplementary data

SI_Schuwirth_2018 SI_Schuwirth_2018

## Footnotes

1 swisstopo (2015): Vector200, TLM; DV 5704 000 000, reproduced with permission of swisstopo/JA100119; Eawag (2014): WWTP of Switzerland: Revised on the basis of the project: Maurer M. und Herlyn A. (2007) Status, costs and investment needs of Swiss wastewater disposal. Eawag/Bafu report (in German).
