# Supplementary material for: Multi-criteria decision analysis for integrated water quality assessment and management support
Source: Water Res X. 2018 Nov 14;1:100010. doi: 10.1016/j.wroa.2018.100010 (PMC6549934; doi:10.1016/j.wroa.2018.100010)
Supplement: SI_Schuwirth_2018 [file mmc1.pdf]

Supplementary material to the paper

Multi-criteria decision analysis for integrated water quality assessment and management support

Nele Schuwirth, Mark Honti, Ivana Logar, Christian Stamm

***Water Resources X*, doi:10.1016/j.wroa.2018.100010**

Contents

1. Water quality assessment ... 2
   1.1. Nutrients ... 2
   1.2. Pesticides ... 2
2. Cost estimation of urban management alternatives ... 6
   2.1. Banning of biocides in façade paints ... 6
   2.2. Increasing the area of permeable pavements ... 9
   2.3. Infiltration of rainwater coming from roofs ... 13
   2.4. Increasing the volume of combined sewer overflow tanks ... 14
   2.5. Upgrading of wastewater treatment plants to remove micropollutants ... 16
3. Cost estimation of agricultural management alternatives ... 19
   3.1. General assumptions ... 19
   3.2. Methods ... 20
   3.3. Parameterization ... 22
   3.4. Arable crops ... 22
   3.5. Grassland ... 24
   3.6. Fruits ... 24
   3.7. Vegetables ... 25
   3.8. Animal production ... 25
   3.9. Set-aside areas ... 26
4. Uncertainty estimation for costs ... 27
5. Absolute costs for management alternatives ... 30
6. R-Script Example Implementation of Value functions ... 31

1. Water quality assessment

1.1. Nutrients

For nutrients, the sampling strategy consists of 12 monthly grab samples. The 90. percentile (C_{90}) of the concentration of all monthly grab samples is evaluated according to Table S 1 (Liechti, 2010).

Table S 1:Nutrient assessment procedure (after Liechti, 2010)

water quality class	PO4 [mg/L P]	NO3 [mg/L N]	NH4 (<10°C) [mg/L N]	NH4 (>10°C) [mg/L N]	value scale	legal require-ments
high	$C_{90} < 0.02$	$C_{90} < 1.5$	$C_{90} < 0.08$	$C_{90} < 0.04$	0.8-1.0	fulfilled
good	$0.02 \le C_{90} < 0.04$	$1.5 \le C_{90} < 5.6$	$0.08 \le C_{90} < 0.4$	$0.04 \le C_{90} < 0.2$	0.6-0.8	
moderate	$0.04 \le C_{90} < 0.06$	$5.6 \le C_{90} < 8.4$	$0.4 \le C_{90} < 0.6$	$0.2 \le C_{90} < 0.3$	0.4-0.6	not fulfilled
poor	$0.06 \le C_{90} < 0.08$	$8.4 \le C_{90} < 11.2$	$0.6 \le C_{90} < 0.8$	$0.3 \le C_{90} < 0.4$	0.2-0.4	
bad	$C_{90} \ge 0.08$	$C_{90} \ge 11.2$	$C_{90} \ge 0.8$	$C_{90} \ge 0.4$	0.0-0.2	

For each water quality parameter, the assessment procedure is translated into a measurable value function according to Langhans et al. (2013), for nutrients by piecewise linear interpolation between class boundaries (Fig. S 1).

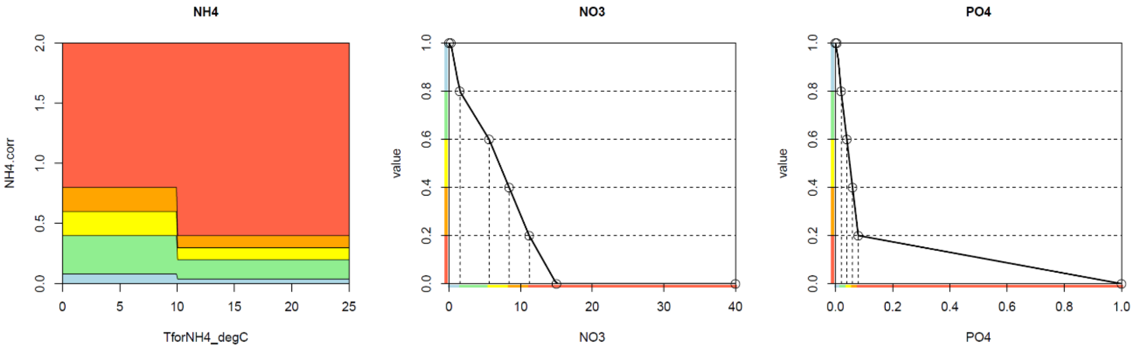

Fig. S 1: Value functions for nutrients

1.2. Pesticides

For pesticides, we adopted the assessment procedure used by the Office for Water, Energy, and Air of the Canton of Zurich (AWEL, 2006). Their sampling strategy consisted of 12 monthly grab samples. The list of pesticides that were analyzed, their mode of action, as well as chronic and acute environmental quality standards (EQS) are given in Table S 2. The chronic and acute risk quotients (concentration of substance *i* in the water sample *j* divided by the chronic and acute EQS of substance *i*, respectively) of substances with same mode of action are summed up.

Supplementary material to Schuwirth, N., Honti, M., Logar, I., Stamm, Ch.: Multi-criteria decision analysis for integrated water quality assessment and management support. *Water Research X*, doi:10.1016/j.wroa.2018.100010

$$RQ_{\text{acute } j} = \sum_i \frac{C_{ij}}{\text{acute } EQS_i}$$

$$RQ_{\text{chronic } j} = \sum_i \frac{C_{ij}}{\text{chronic } EQS_i}$$

Afterwards, for each mode of action group, the quality classes are determined according to Table S 3.

Table S 2: Pesticides considered including their proposed environmental quality standards (EQS) from website of the Swiss Ecotox Centre, http://www.ecotoxcentre.ch/expert-service/quality-standards/proposals-for-acute-and-chronic-quality-standards/ accessed 18.09.2013:**

Substance	Class	Mode of action*	Chronic eqs ng/L	Acute eqs ng/L
Atrazine†	Herbicide	PhotosynthInh	600	2000
Diuron	Herbicide	PhotosynthInh	20	60
Isoproturon	Herbicide	PhotosynthInh	320	1200
Terbutryn	Herbicide	PhotosynthInh	65	340
Terbuthylazine	Herbicide	PhotosynthInh	220	1280
2,4-D	Herbicide	AuxinAct	200	1300
MCPA	Herbicide	AuxinAct	1340	15200
Mecoprop	Herbicide	AuxinAct	3600	187000
S-Metolachlor	Herbicide	VLCFASI	270	2700
Diazinon	Insecticide	AChEI	15	15
Pirimicarb	Insecticide	AChEI	90	1600

†: no longer registered in Switzerland.

*AChEI: acetylcholinesterase inhibitors, organophosphates, VLCFASI: very-long-chain fatty acid synthesis inhibitors, chloroacetanilides, AuxinAct: herbicides that influence the auxin activity of specific plants, PhotosynthInh: photosynthesis inhibitors affecting Photosystem II

**Note that the proposals for EQS were still subject to changes during the course of this study. The current chronic and acute EQS values in ng/L have changed for Diuron (70, 250), Isoproturon (640, 1700), 2,4-D (600, 4000), MCPA (660, 6400), Metolachlor (690, 3300), Diazinon (12, 20), Pirimicarb (90, 1800) (accessed 9.5.2018).

Table S 3: Assessment procedure for pesticides (AWEL, 2006)

water quality class		value scale
high	$RQ_{\text{chronic}\,j}$ of all water samples j are < 0.5	1
good	max($RQ_{\text{chronic}\,j}$) ≥ 0.5 and<br>number of ($RQ_{\text{chronic}\,j}$ ≥ 1) < 3 within two years, but not twice in a row	0.75
moderate	max($RQ_{\text{chronic}\,j}$) ≥ 1 and<br>number of ($RQ_{\text{chronic}\,j}$ ≥ 1) ≥ 3 or RQ_{chronic} ≥ 1 twice in a row	0.5
poor	$RQ_{\text{chronic}\,j}$ ≥ 1 for more than half of the samples j	0.25
bad	max($RQ_{\text{acute}\,j}$) ≥ 1	0

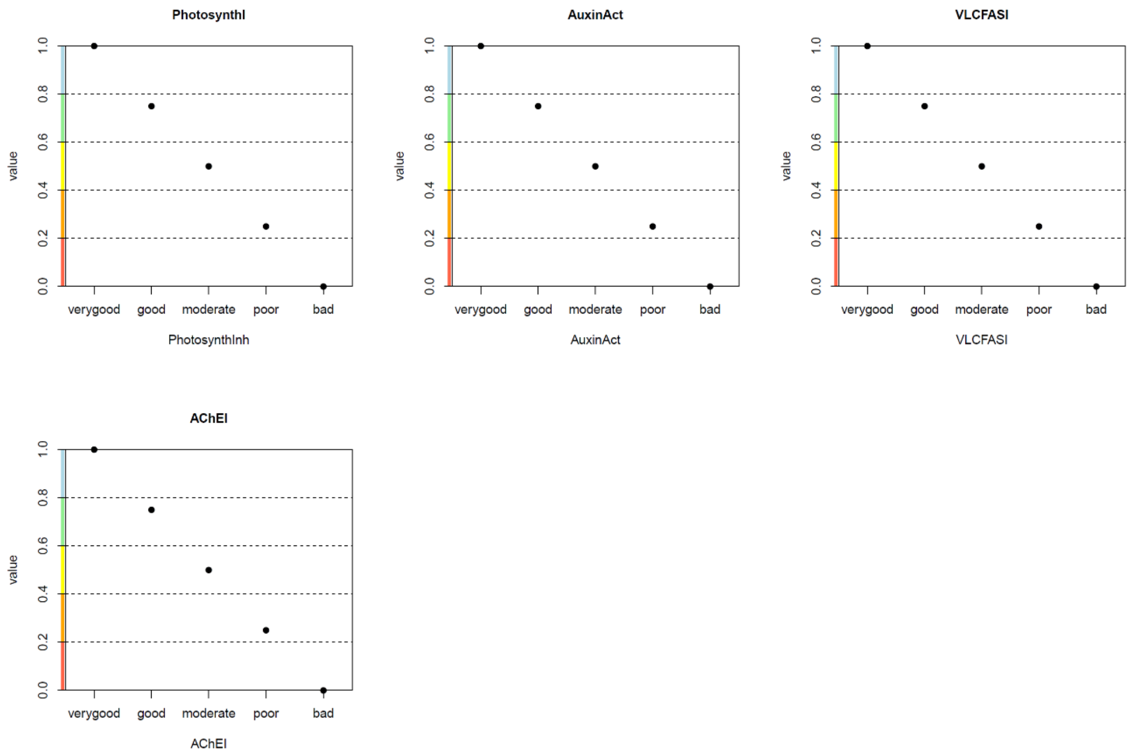

Fig. S 2: Value functions for pesticide groups.

References:

Awel. (2006) Wasserqualität der Seen, Fliessgewässer und des Grundwasser im Kanton Zürich, Statusbericht 2006. (Ed W. Awel Amt Für Abfall, Energie Und Luft.), Zürich.

Langhans S.D., Lienert J., Schuwirth N. & Reichert P. (2013) How to make river assessments comparable: A demonstration for hydromorphology. *Ecological Indicators,* **32,** 264-275.

Langhans S.D., Reichert P. & Schuwirth N. (2014) The method matters: A guide for indicator aggregation in ecological assessments. *Ecological Indicators,* **45,** 494-507.

Supplementary material to Schuwirth, N., Honti, M., Logar, I., Stamm, Ch.: Multi-criteria decision analysis for integrated water quality assessment and management support. *Water Research X*, doi:10.1016/j.wroa.2018.100010

Liechti P. (2010) Methoden zur Untersuchung und Beurteilung der Fliessgewässer. Chemisch-physikalische Erhebungen, Nährstoffe. Umwelt-Vollzug Nr. 1005. Bundesamt für Umwelt, Bern. 44 S. .

Supplementary material to Schuwirth, N., Honti, M., Logar, I., Stamm, Ch.: Multi-criteria decision analysis for integrated water quality assessment and management support. *Water Research X*, doi:10.1016/j.wroa.2018.100010

2. Cost estimation of urban management alternatives

The costs of all management alternatives are estimated on an annual basis. Uncertainty estimation is described in section 4.Banning of biocides in façade paints

This management alternative assumes that the use of biocides is prohibited in façade paints. The costs of banning the use of biocides in façade paints (C) are calculated as the difference between the costs of painting the façades in Mönchaltorfer Aa study area with paints free of biocides $(C_{no\ bio})$ and the costs of painting the same façade area with paints that contain biocides (C_{bio}):

$$C = C_{no\ bio} - C_{bio}\ .$$

$C_{no\ bio}$ and C_{bio} are further defined by the unit costs $\left(C_{u_i}\right)$, i.e. the costs of painting one m^2 of a façade, the façade area (A) and the lifespan (L_i) of each paint type i $(i = no\ bio, bio)$. Therefore, the costs of painting the façades in the case study area with paints free of biocides are calculated as follows:

$$C_{no\ bio} = C_{u\ no\ bio} \times \frac{A}{L_{no\ bio}}\ ,$$

while the costs of painting the façades with paints containing biocides are estimated according to the formula:

$$C_{bio} = C_{u\ bio} \times \frac{A}{L_{bio}}\ .$$

The expected future façade areas under four scenarios are estimated based on the data from the Sustainable Water Infrastructure Planning (SWIP) project at Eawag, which is then adjusted to the Mönchaltorfer Aa study area. The assumed façade areas in future scenarios are listed in Table S 4.

Table S 4: Assumed façade surface in the present and four future scenarios

Scenario	Façade surface (m^2)
Present	1.457.560
Status quo	1.457.560
Decline	1.457.560
Exploding growth	9.739.808
Moderate growth	1.685.388

Unit costs of painting a façade ($C_{no\ bio}$ and C_{bio}) were calculated based on the offers made for the job advertisement for painting a façade in Switzerland (www.ofri.ch/jobs/6295; assessed 17 July 2013). As the best cost estimate, we have used the average of a total of 15 different offers, resulting in the unit cost of 31.47 CHF/m^2. The minimum and the maximum estimates of unit costs correspond to the lowest and the highest offered amounts for performing this job (i.e. 20.67 CHF/m^2 and 38.13 CHF/m^2, respectively). The unit cost of painting a façade usually consists of the cost of human labor needed for performing the painting activity and the cost of paint:

$$C_{u_i} = C_{u\ labor} + C_{u_i\ paint} \ .$$

However, most of the online offers for painting the facade do not specify the type of paint to be used, which implies that our unit costs already include both cost elements and are thus equal for the paints with and without biocides. In order to distinguish between the costs of painting the façade with paints containing and those that do not contain biocides, we have also collected data on the prices of the two paint types. Caparol (2013) price list served as a data source for estimating the costs of façade paints containing biocides, while the prices of Keim (personal communication) was used as an indicator of costs of façade paint free of biocides. Given that each paint type includes a number of paints with varying prices, the average of all paint prices was used as the best estimate of the cost of paint, while the lowest and the highest paint prices served as the minimum and maximum cost estimates of paint. The resulting cost estimates are listed in Table S 5.

Table S 5: Prices of façade paints with and without biocides

Paint type (i)	Paint prices		
	Minimum estimate	Best estimate	Maximum estimate
$no\ bio$	10.2 CHF/kg	14.1 CHF/kg	17.95 CHF/kg
bio	8.5 CHF/kg	11.62 CHF/kg	14.5 CHF/kg

To estimate the unit cost of paint $\left(C_{u_i\ paint}\right)$ we multiply the paint prices by the amount of paint needed per m^2. For this purpose, we assume that for both paint types two layers of paint are always applied, which corresponds to approximately 400g of paint per m^2. The difference between the unit cost of paint with and without biocides is then used as a variance around the unit cost of painting a façade (including both labor and paint costs) to derive separate unit cost of painting a façade with paints containing biocides and those that are free of biocides. These two unit cost estimates are shown in Table S 6. This table also presents the assumed lifespan of the paints with and without biocides, which are obtained from BBSR (2011).

doi:10.1016/j.wroa.2018.100010

Table S 6: Unit costs and lifespan of painting façades with and without biocides

Paint type (i)	Unit costs (C_{u_i})			Lifespan (L_i)
	Minimum estimate	Best estimate	Maximum estimate	
$no\ bio$	21.01 CHF/m^2	31.97 CHF/m^2	38.82 CHF/m^2	15 years
bio	20.33 CHF/m^2	30.97 CHF/m^2	37.44 CHF/m^2	20 years

Note that there are several additional sources of uncertainty, which are not accounted for in these cost estimates. For example, the amount of paint that needs to be applied to a façade depends on the type of façade, while the frequency of painting depends on the location of the building because it might be influenced by local climate conditions (e.g., humidity level), traffic intensity, and similar external factors. Although most commonly two layers of paint are applied, this may not always be the case. For the purpose of estimating these costs, we have used paint prices of two specific producers, but the paint prices can vary depending on the producer. Finally, predictions about the lifespans of paints are subject to substantial variations.

2.2. Increasing the area of permeable pavements

In this urban water management alternative three different pavement types are considered, namely standard impermeable pavement (pavement type a), concrete tiles with permeable grout (pavement type b), and concrete grid blocks with grass (pavement type c). The costs of increasing urban permeable surfaces under four future scenarios are estimated based on the assumptions made about the type of pavements that would be used in the future and their expected future surfaces $(A_i;\ i = a,b,c)$. These assumptions are presented in Table S 7.

Table S 7: Present and assumed future pavement types and their surfaces for the management alternative under each scenario

Scenario	Sidewalks	Parking lots	Paved house surroundings
Present	100% type *a* (17.6 ha)	100% type *a* (10.3 ha)	100% type *a* (130 ha)
Status quo	100% type *b* (17.6 ha)	50% type *b* (5.15 ha), 50% type *c* (5.15 ha)	100% type *c* (130 ha)
Decline	100% type *b* (17.6 ha)	50% type *b* (5.15 ha), 50% type *c* (5.15 ha)	100% type *c* (130 ha)
Exploding growth	100% type *a* (69 ha)	100% type *b* (40.2 ha)	100% type *a* (506 ha)
Moderate growth	100% type *b* (18.5 ha)	50% type *b* (5.45 ha), 50% type *c* (5.45 ha)	100% type *c* (136 ha)

The annual costs of increasing the area of permeable pavements are defined as the difference between the costs of implementing this management alternative (C_{alt}) and the costs of not implementing the alternative ($C_{no\ alt}$):

$$C = C_{alt} - C_{no\ alt} \ .$$

The costs of implementing the alternative are further specified as follows:

$$C_{alt} = \frac{C_a \times A_a}{L_a} + \frac{C_b \times A_b}{L_b} + \frac{C_c \times A_c}{L_c} \ ,$$

where C_{u_i} denote unit costs (i.e. costs per m^2) of each pavement type i $(i = a, b, c)$, A_i represents absolute future surface of each pavement type expressed in m^2, and L_i is the expected lifespan of each pavement type, measured in years. Since not implementing this management alternative implies that the standard impermeable pavement (i.e. pavement type a) would be used for the entire future surface, the associated costs of not implementing the alternative are calculated as follows:

$$C_{no\ alt} = \frac{C_a \times (A_a + A_b + A_c)}{L_a} .$$

The same cost calculation procedure was used for obtaining minimum, best and maximum cost estimates. Data on unit costs (C_{u_i}) and lifespan of pavement types (L_i) are shown in Table S 8. Unit costs comprise both material and construction costs of pavements. In addition to the minimum and maximum cost ranges, the additional sources of uncertainty concerning cost estimates include the type of soil on which the pavement is placed, whether the material is bought on the open market or through fair trade, and the size of concrete tiles or blocks used. Lifespan, on the other hand, can vary depending on the intensity of the traffic.

Table S 8: Unit costs and lifespan of different pavement types

Pavement type (i)	Unit costs (C_{u_i})			Lifespan (L_i)
	Minimum estimate	Best estimate	Maximum estimate	
a	34 CHF/m^2	42 CHF/m^2	55 CHF/m^2	15 years
b	55 CHF/m^2	75 CHF/m^2	90 CHF/m^2	20 years
c	85 CHF/m^2	95 CHF/m^2	115 CHF/m^2	20 years

Source: Fachverband Infra (personal communication).

2.3. Infiltration of rainwater coming from roofs

For this urban water management alternative we assume that the share of roof area connected to infiltration will increase from the current 5% to 20% in all future scenarios. The predicted total roof areas in the future scenarios and corresponding roof areas connected to infiltration are shown in Table S 9. A distinction is made between roof area that would be connected to infiltration without implementing this management alternative, $A_{no\ alt}$ (i.e. 5% of the total future roof area) and roof area that would be connected to infiltration in the case that this alternative is implemented, A_{alt} (20% of the total future roof area). The difference between the two represents the additional roof area that would be infiltrated if the alternative was implemented:

$$\Delta A = A_{alt} - A_{no\ alt} \,.$$

Table S 9: Assumed roof area in future scenarios and roof areas connected to infiltration with and without the management alternative

Scenario	Total roof area (m^2)	Roof area connected to infiltration without alternative $(A_{no\ alt})$	Roof area connected to infiltration with alternative (A_{alt})	Difference in roof area connected to infiltration with and without alternative (ΔA)
Present	1.360.000	68.000	68.000	0
Status quo	1.360.000	68.000	272.000	204.000
Decline	1.360.000	68.000	272.000	204.000
Exploding growth	5.300.000	265.000	1.060.000	795.000
Moderate growth	1.420.000	71.000	284.000	213.000

We further assume that only investment costs exist and that there are no operating costs associated with infiltration of rainwater coming from roofs. Best, maximum and minimum estimates of the unit costs, C_u (i.e. investment costs per m^2) as well as the lifespan of the investment (L) are presented in Table S 10. Both unit cost and lifespan data is obtained from Fritsche (2013) and Katrin (2011).

Table S 10: Unit costs and lifespan of infiltrating rainwater coming from roofs

Unit costs (C_u)			Lifespan (L)
Minimum estimate	Best estimate	Maximum estimate	
1.5 CHF/m^2	26 CHF/m^2	84 CHF/m^2	20 years

The total annual cost (C) of implementing this urban water management alternative is then calculated with the formula:

$$C = \frac{C_u}{L} \times \Delta A .$$

The same calculation procedure has been used for deriving best, maximum and minimum annual cost estimates of this management alternative.

2.4. Increasing the volume of combined sewer overflow tanks

We assume that the volume of combined sewer overflow (CSO) tanks increases by 100% under all future scenarios. The existing CSO tanks $(i = 1, 2, ..., 12)$ in the Mönchaltorfer Aa basin and their current respective volumes (V_i) are presented in Table S 11. Uncertainty about the current volumes of existing tanks is approximately +/-10%.

Table S 11: Current CSO tanks in the case study area and their volumes

Name of CSO tank	Municipality	Current effective volume (m^3)
Kirchwies	Egg	88
Oberesslingen	Egg	489
RB_ARA	Egg	216
RB_Aspholz 1	Egg	210
RB_Aspholz 2 & 3	Egg	590
RB_Berghof	Gossau	135
RB_Unterhofen	Gossau	400
RB_ARA	Gossau	225
RB_Bueel	Grüningen	300
RKB_ARA	Mönchaltorf	400
RKB_Weibelacher	Mönchaltorf	115
FB_Esslingerstrasse	Mönchaltorf	200
**TOTAL**		**3368**

The costs of increasing the volumes of CSO tanks consist of the costs of constructing the concrete part of the tank and the equipment costs. We assume that there are no operating costs associated with this management alternative since these costs are not expected to be substantial. The unit costs (i.e. costs per m^3 of a CSO tank) related to both construction $\left(C_{u_i\ constr}\right)$ and equipment $\left(C_{u_i\ equip}\right)$ vary with the tank volume due to the economies of scale. The unit costs are calculated using data provided in HBT (2012) on construction and equipment costs of five CSO tanks with different volumes. That is, the best estimates of construction unit costs and equipment unit costs are calculated based on the function that is derived from these five data points. The resulting functions are presented in Fig. S 3. for construction unit costs and equipment unit costs.

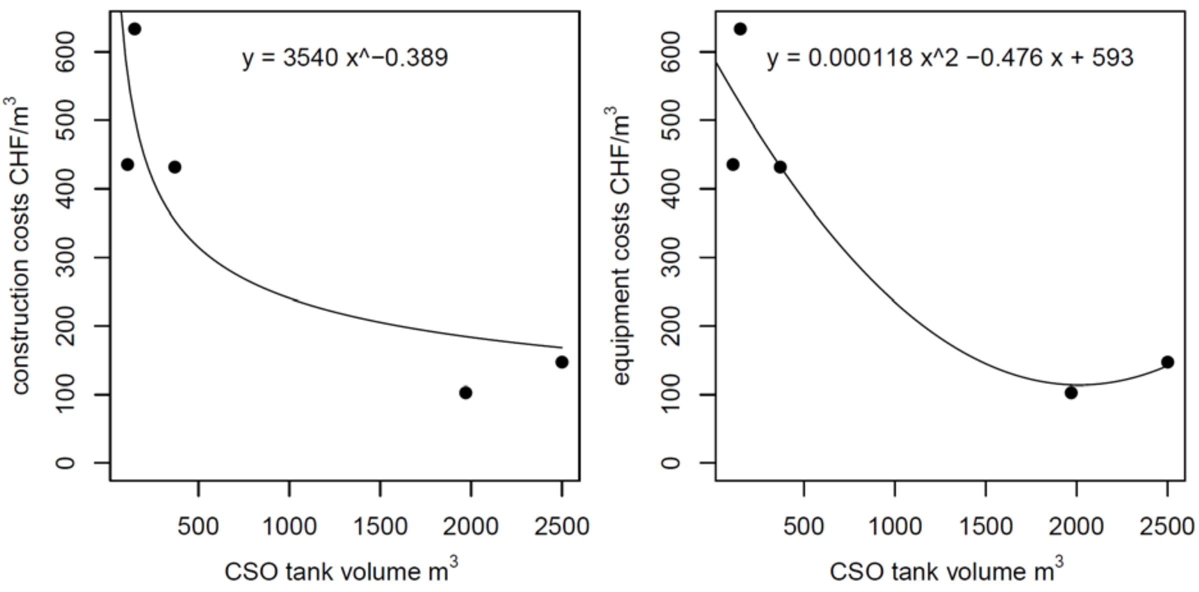

Fig. S 3: Function for estimating unit costs (best estimate) of constructing CSO tanks (left panel) and of equipment for CSO tanks (right panel).

The minimum construction and equipment unit cost estimates equal the lowest unit costs out of these five CSO tanks, while the maximum unit cost estimates correspond to the tank with the highest unit costs. Finally, the lifespan of the concrete part of the CSO tanks is assumed to be 40 years, while the equipment is assumed to last for 17.5 years. All unit costs and lifespan data used for calculating the costs of increasing the volume of CSO tanks are shown in Table S 12.

Supplementary material to Schuwirth, N., Honti, M., Logar, I., Stamm, Ch.: Multi-criteria decision analysis for integrated water quality assessment and management support. *Water Research X*, doi:10.1016/j.wroa.2018.100010

Table S 12: Unit costs and lifespan of a CSO tank

Unit costs of constructing the concrete part of a CSO tank $\left(C_{u_i\ constr}\right)$			Lifespan $\left(L_{constr}\right)$
**Minimum estimate**	**Best estimate**	**Maximum estimate**	
948 CHF/m³	$C_{u_i\ \mathrm{constr}} = 3540 \cdot V_i^{-0.389}$	6164 CHF/m³	40 years
**Unit costs of the equipment for CSO tank** $\left(C_{u_i\ equip}\right)$			**Lifespan** $\left(L_{equip}\right)$
**Minimum estimate**	**Best estimate**	**Maximum estimate**	
104 CHF/m³	$C_{u_i\ \mathrm{equip}} = 0.000118 \cdot V_i^2 - 0.476 \cdot V_i + 593$	633 CHF/m³	17.5 years

The total annual costs (C) of increasing the volumes of CSO tanks are then calculated as follows:

$$C = \sum_{i=1}^{12} \left(\frac{C_{u_i\ constr}}{L_{constr}} + \frac{C_{u_i\ equip}}{L_{equip}} \right) \times V_i \ .$$

2.5. Upgrading of wastewater treatment plants to remove micropollutants

The assumption made for this management alternative is that the three wastewater treatment plants (WWTPs) which exist in the Mönchaltorfer Aa basin will be upgraded with the purpose of removing micropollutants from wastewater in the treatment process. The costs of the upgrades depend mainly on the capacity (size) of a WWTP, which is usually expressed in terms of person equivalent (PE). Capacities of each WWTP i $(i = 1, 2, 3)$ in the case study area under future scenarios were determined proportionally to the assumed population growth in each scenario. Current and predicted capacities in 2050 for the three WWTPs in Mönchaltorfer Aa are listed in Table S 13.

Table S 13: Present and assumed future capacities of WWTPs in Mönchaltorfer Aa

Scenario	Egg-Oetwil $(i=1)$	Gossau $(i=2)$	Mönchaltorf $(i=3)$
Present	13.000 PE	15.000 PE	4.000 PE
Status quo	13.000 PE	15.000 PE	4.000 PE
Doom	13.000 PE	15.000 PE	4.000 PE
Exploding growth	107.640 PE	124.200 PE	33.120 PE
Moderate growth	15.600 PE	18.000 PE	4.800 PE

The report by Hunziker Betatech AG (2008) was the main source of cost data for this management alternative. This report presents the cost estimates of upgrading six different WWTPs in Switzerland with varying capacities. We have estimated the cost functions for investment and operating costs based on the six data points provided in the report, which indicate WWTP capacities and associated costs (Fig. S 4).

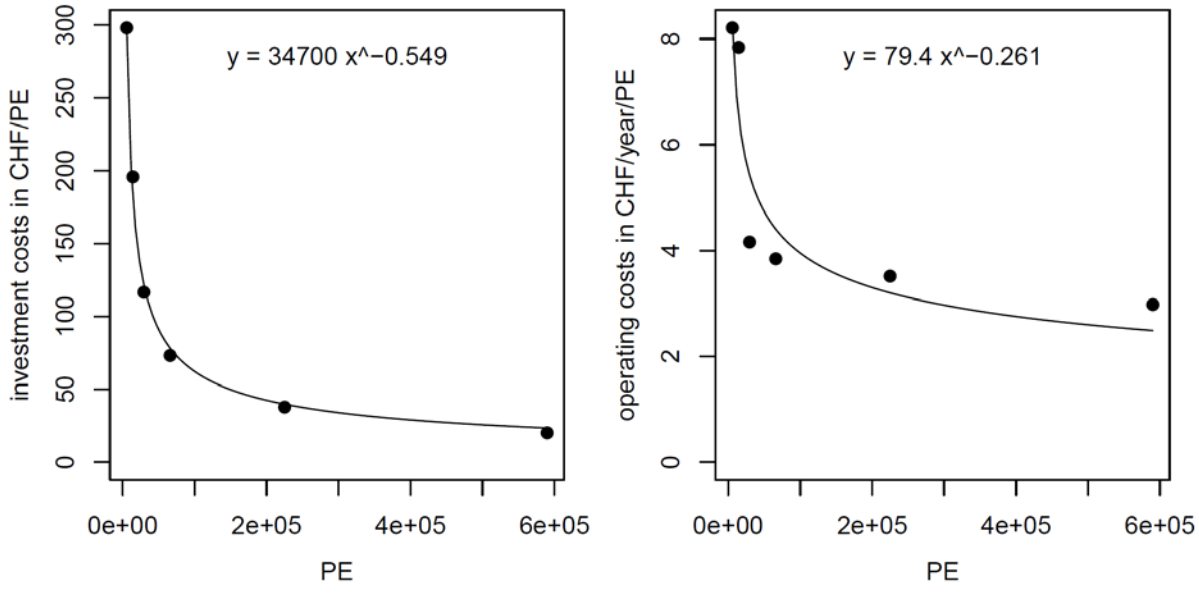

Fig. S 4: Functions for estimating investment (left panel) and operating (right panel) costs of upgrading WWTPs based on their size expressed as person equivalents (PE).

The additional investment and operating costs of upgraded WWTPs are hence estimated based on the function

$C_{\text{invest}\,i} = 34743\,PE^{-0.549}$

$C_{\text{oper}\,i} = 79.4\,PE^{-0.261}$

where *PE* are person equivalents.

The annual costs of upgrading each WWTP in the case study area (C_i) are calculated as follows:

$$C_i = C_{\text{invest}\,i}\,PE\left(\frac{f_{\text{constr}}}{L_{\text{constr}}} + \frac{1 - f_{\text{constr}}}{L_{\text{equip}}}\right) + C_{\text{oper}\,i}\,PE$$

where C_{invest_i} denotes investment costs into the upgrades of a WWTP i, L_{constr} is the lifespan of the investment regarding construction, which is assumed to be 30 years for all WWTPs, L_{equip} is the lifespan of the investment regarding equipment, which is assumed to be 12 years for all WWTPs and f_{constr} is the fraction of investment costs for construction, which is assumed to be 50%, C_{oper_i} represents annual operating costs of each WWTP.

Finally, the annual costs of individual WWTPs are summed up in order to derive the total annual costs (C) of implementing this urban water management alternative in Mönchaltorfer Aa basin:

$$C = \sum_{i=1}^{3} C_i \,.$$

References:

BBSR - Das Bundesinstitut für Bau-, Stadt- und Raumforschung (2011). Nutzungsdauern von Bauteilen fur Lebenszyklusanalysen nach Bewertungssystem Nachhaltiges Bauen. http://www.nachhaltigesbauen.de/baustoff-und-gebaeudedaten/nutzungsdauern-von-bauteilen.html

Caparol (2013). Baumaler Preisliste 1/2013. http://www.caparol.ch/Portals/_ch/upload/images/service/Preisliste%20%2001_2013_web.pdf.

Fritsche, C. (2013). Costs of the Waste Water Disposal in Switzerland. Internal Eawag report.

HBT (2012). Kostdaten Sammlung SWIP - Dokumentation. Hunziker Betatech AG, Winterthur.

Hunziker Betatech AG (2008). Massnahmen in ARA zur weitergehenden Elimination von Mikrovereinigungen: Kostenstudie. Swiss Federal Office for the Environment, Bern.K

Katrin (2011). Zusammenstellung Versickerung, Grauwasserbehandlung, dezentrale Anlagen. Bericht Eawag, Dubendorf.

3. Cost estimation of agricultural management alternatives

3.1. General assumptions

It is assumed that specific management alternatives are declared mandatory within the study area. The costs (C) related to the agricultural management alternatives (a) are mainly caused by

i) a decrease in agricultural income ($In_a < In_{ref}$) and in workload ($W_a \leq W_{ref}$) of the farmers compared to the reference situation (see below). The agricultural income is treated as the farmers` revenue minus the production costs and including federal direct payments. We assume that the loss in income is compensated by public funding that is paid in addition to existing federal direct payments (a form of subsidies).

ii) an increase in workload ($W_a > W_{ref}$), which decreases the productivity of farming. It is assumed that only the reference workload can be accomplished by the farmer, which results in a loss of income in case a potential higher income does not compensate for the additional working hours ($In_a < In_{ref} \times \frac{W_a}{W_{ref}}$). This is true also in the case the farmer would employ another person for the additional working hours, which would increase the labour expenses.

The costs corresponding to situation i) are calculated in a similar way as done by the federal authorities for compensation paid projects according to article 62a of the Swiss Waters Protection Act based on standard values for 2012 (AGRIDEA and FiBL, 2012). For some vegetable crops we based the estimates on specific literature (Möhring et al., 2012). Basically, we calculate the contribution margin of the reference situation (= current land use and income In_{ref}) and subtract the contribution margin of the target situation (In_a).

The costs corresponding to situation ii) are calculated by assuming that the average productivity per working hour is constant and that the same agricultural area is cultivated. These costs are calculated by multiplying the workload in the reference situation (W_{ref}) by the loss in agricultural income earned per working hour in the selected alternative. Overall, these two situations are expressed as follows:

$$C_a = \begin{cases} \max\left(0, In_{ref} - In_a\right) & W_a \leq W_{ref} \\ \max\left(0, In_{ref} - In_a \times \frac{W_{ref}}{W_a}\right) & W_a > W_{ref} \end{cases} \quad (1)$$

For illustration of these different situations, see Fig. S 5 (i) and (ii).

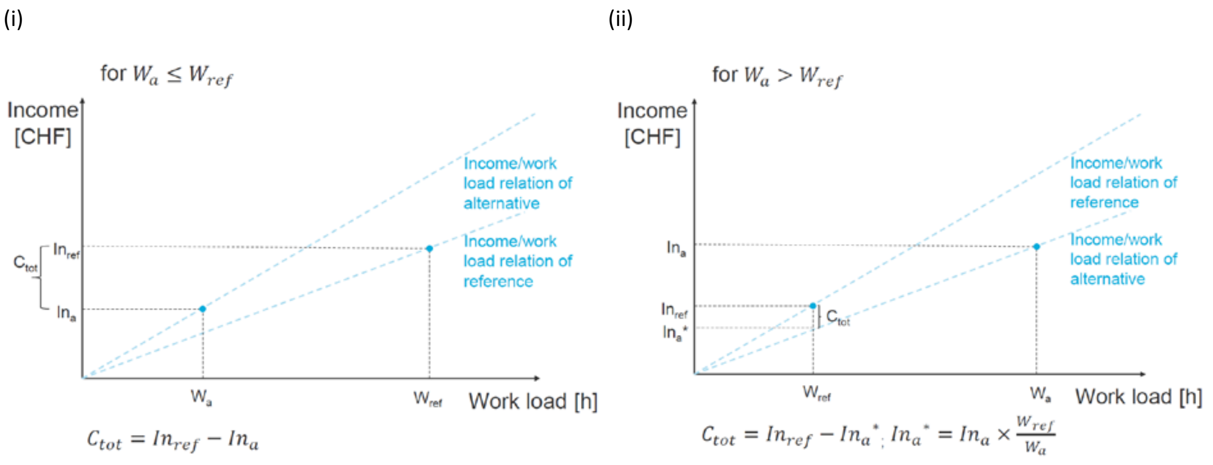

Fig. S 5: *Graphical illustration of case (i)* $W_a \le W_{ref}$ *(for* $In_a < In_{ref}$ *; otherwise the costs are zero) and case (ii)* $W_a > W_{ref}$ *(for* $In_a < In_{ref} \times \dfrac{W_a}{W_{ref}}$ *; otherwise the costs are zero)*

3.2. Methods

Here, we assume that we know the distribution of the agricultural crops and the animal stocking density under current conditions. The total agricultural area is the sum of areas of a finite set of major crops:

$$A_{agro} = A_{agro\_ref} \times \sum_i \frac{A_i}{A_{agro\_ref}} = A_{agro\_ref} \times \sum_i a_i \qquad (2)$$

where A_{agro} is the total agricultural area for a scenario, A_{agro_ref} is the total agricultural area for the reference conditions, and a_i are the fractions of each crop on the total agricultural acreage.

The total number of dairy cow equivalents DEq_{tot} is given as follows:

$$DEq_{tot} = A_{agro\_ref} \times \sum_i \frac{dqe_i \times N_i}{A_{agro\_ref}} = A_{agro\_ref} \times \sum_i dqe_i \, n_i \qquad (3)$$

where dqe_i represents the dairy cow equivalents on an individual of a given animal species. The dairy cow equivalent is a unit for the animal production, where the weight of each species is measured in comparison with an adult dairy cow. Hence, one adult dairy cow equals 1 DEq, one sheep equals 0.1 DEq, for example.

We assume that the relative coverage of each crop a_i (=$\dfrac{A_i}{A_{agro\_ref}}$) and the relative stocking density of animal production n_i (=$\dfrac{N_i}{A_{agro\_ref}}$) are the same for the reference and the organic farming management alternatives and remain unchanged for the future scenarios. This holds almost true for

the buffer strip alternative: here we assume that the distribution remains constant on those areas not converted into riparian hedges:

$$A_{agro\_buf} = A_{agro\_buf\_tot} - A_{riparian} \tag{4}$$

For the Nature Park alternative we assume that all land is used as extensive permanent grassland. Accordingly, we assume that stocking densities are at a very low level (0.5 dairy cow equivalents per ha) and that there is no dairy farming anymore.

Income

The absolute agricultural income for each alternative is then calculated as the sum of income generated by crop and animal production times the overall agricultural area of the alternative (A_{agro_scen}) as follows:

$$In_{tot\_scen} = A_{agro\_scen} \times \left(\sum_i a_i \times cm_i + \sum_j n_j \times cm_j \right) \tag{5}$$

where cm is the average contribution margin, the difference between overall revenue of a period and variable costs of this period, of a given crop *i* and animal *j*, respectively. This value (cm) is calculated as the sum of fractions of contribution margins for conventional (= ÖLN farms, ÖLN stands for "ökologischer Leistungsnachweis") and organic farms (f_{org}). Because for each crop *i* one may have to consider several sub-types, the contribution margin is calculated as follows:

$$cm_i = f_{org} \times \sum_k cm_k^{org} + (1 - f_{org}) \times \sum_k cm_k^{non-org} \tag{6}$$

where *k* stands for the crop and animal sub-types.

Workload

The total workload in the agricultural sector for each management alternative is calculated in an analogous way as follows:

$$W_{tot\_scen} = A_{agro\_scen} \times \left(\sum_i a_i \times w_i + \sum_i n_i \times w_i \right) \tag{7}$$

where w_i is the average workload per unit area of a given crop or per animal of type *i*. This value is calculated as the sum of the fractions of workload contributed by conventional (= ÖLN farms) and organic farms. Because for each crop one may have to consider several sub-types, w_i is calculated as follows:

$$w_i = f_{org} \times \sum_j w_j^{org} + (1 - f_{org}) \times \sum_j w_j^{non-org} \tag{8}$$

Supplementary material to Schuwirth, N., Honti, M., Logar, I., Stamm, Ch.: Multi-criteria decision analysis for integrated water quality assessment and management support. *Water Research X*, doi:10.1016/j.wroa.2018.100010

3.3. Parameterization

Here we assume the following crops including the sub-types to be present and we distinguish among them for calculating the contribution margins cm and the specific workload w (the list is based mainly on (AGRIDEA and FiBL, 2012) with reference to (Möhring et al., 2012) for vegetables).

The parameter values (fractions of production types, all between 0 and 1), mainly for crops, not for animal production, could be taken from FOAG data (FOAG 2012) from the 5 municipalities mostly contributing to the area of the catchment of the Mönchaltorfer Aa (Oetwil am See, Egg, Mönchaltorf, Grüningen, Gossau (ZH)). For those parameters a relative small uncertainty was assumed (relative sd= 0.1). For only coarsely estimated parameters, the uncertainty was assumed to be larger (relative sd= 0.2 or 0.25). In the data from FOAG, some areas are double-counted, because fields can be assigned to both, “organic farming” and “extensive production”. We counted those fields as “organic”, assuming that they are farmed in an organic way, in case they are registered as both. For parameters for which this assumption actually makes a difference in the fraction for the production type, a medium uncertainty was assigned (relative sd= 0.15). From the FOAG data, crop-specific fractions of organic production could be calculated. For those crops and animal products, for which data was missing, the overall fraction of organic farming (f_{org}) was used.

Mathematically, the contribution margins cm and the specific workload w are described by the same equations.

3.4. Arable crops

Cereals (represented by 4 crops making up > 94.9% of the cereal production in CH (with corn excluded) based on BFS statistics:
http://www.bfs.admin.ch/bfs/portal/de/index/themen/07/03/blank/data/01/04.Document.21039.xls.

Because winter wheat is currently the dominant form, we have neglected summer wheat (http://www.news.admin.ch/message/index.html?lang=de&print_style=yes&msg-id=30251).

- Winter wheat (WW Top ÖLN(intensive)/Bio Grosshandel; `cmWW-OLN/ cmWW-bio`)
- Dinkel (spelt) (ÖLN intensiv Grosshandel)/Bio Grosshandel; `cmDi-OLN/cmDi-bio`)
- Triticale (Tri ÖLN(intensive)/Bio Grosshandel; `cmTri-OLN/ cmTri-bio`)
- Barley (Bar ÖLN(intensive)/Bio Grosshandel; `cmBar-OLN/ cmBar-bio`)

For those 4 cereal types, we estimated an organic farming fraction and a conventional (i.e. non-organic) farming fraction, which was further divided into intensive and extensive farming fractions. For wheat we considered both, bread wheat (“Brotgetreide”) and feed wheat (“Futtergetreide”), separately.

Based on this information the contribution margins cm_i of cereals are calculated as follows:

$$cm_{wheat} = f_{org\_wheat} cm_{wheat}^{org} + (1 - f_{org\_wheat})\left(f_{wheat\_int} cm_{wheat\_int}^{non-org} + (1 - f_{wheat\_int}) cm_{wheat\_ext}^{non-org}\right)$$

(This equation is the same for feed wheat and normal winter wheat)

Supplementary material to Schuwirth, N., Honti, M., Logar, I., Stamm, Ch.: Multi-criteria decision analysis for integrated water quality assessment and management support. *Water Research X*, doi:10.1016/j.wroa.2018.100010

$$cm_{dinkel} = f_{org\_dinkel} cm_{dinkel}^{org} + \left(1 - f_{org\_dinkel}\right)\left(f_{dinkel\_\mathrm{int}} cm_{dinkel\_\mathrm{int}}^{non-org} + \left(1 - f_{dinkel\_\mathrm{int}}\right) cm_{dinkel\_ext}^{non-org}\right)$$

$$cm_{tri} = f_{org\_tri} cm_{tri}^{org} + \left(1 - f_{org\_tri}\right)\left(f_{tri\_\mathrm{int}} cm_{tri\_\mathrm{int}}^{non-org} + \left(1 - f_{tri\_\mathrm{int}}\right) cm_{tri\_ext}^{non-org}\right)$$

$$cm_{barley} = f_{org\_barley} cm_{barley}^{org} + \left(1 - f_{org\_barley}\right)\left(f_{barley\_\mathrm{int}} cm_{barley\_\mathrm{int}}^{non-org} + \left(1 - f_{barley\_\mathrm{int}}\right) cm_{barley\_ext}^{non-org}\right)$$

- Corn is represented by “Silomais getrocknet, Grosshandel” and by “Körnermais Grosshandel”. For the non-organic production system two intensity levels are distinguished (with f_{corn_int} being the fraction of intensive production). Accordingly, the contribution margin cm_{corn} is calculated as follows:

$$cm_{corn\_silage} = f_{org\_corn\_silage}\, cm_{corn\_silage}^{org} + \left(1 - f_{org\_corn\_silage}\right)\begin{pmatrix} f_{corn\_silage\_\mathrm{int}} cm_{corn\_silage\_\mathrm{int}}^{non-org} + \\ \left(1 - f_{corn\_silage\_\mathrm{int}}\right) cm_{corn\_silage\_ext}^{non-org} \end{pmatrix}$$

$$cm_{corn\_grain} = f_{org\_corn\_grain}\, cm_{corn\_grain}^{org} + \left(1 - f_{org\_corn\_grain}\right)\begin{pmatrix} f_{corn\_grain\_\mathrm{int}} cm_{corn\_grain\_\mathrm{int}}^{non-org} + \\ \left(1 - f_{corn\_grain\_\mathrm{int}}\right) cm_{corn\_grain\_ext}^{non-org} \end{pmatrix}$$

- Potatoes are represented by “Speisekartoffeln Grosshandel” and “Veredelungskartoffeln Grosshandel” with f_{pot_direct} giving the respective fractions of “Speisekartoffeln Grosshandel”. For the non-organic production system two intensity levels are distinguished (with f_{pot_int} being the fraction of intensive production). Accordingly, the contribution margin cm_{pot} is calculated as follows:

$$\begin{aligned} cm_{pot} &= f_{org\_pot}\left(f_{pot\_direct} cm_{pot\_direct}^{org} + \left(1 - f_{pot\_direct}\right) cm_{pot\_up}^{org}\right) \\ &+ \left(1 - f_{org\_pot}\right) f_{pot\_\mathrm{int}}\left(f_{pot\_direct} cm_{pot\_direct\_\mathrm{int}}^{non-org} + \left(1 - f_{pot\_direct}\right) cm_{pot\_up\_\mathrm{int}}^{non-org}\right) \\ &+ \left(1 - f_{org\_pot}\right)\left(1 - f_{pot\_\mathrm{int}}\right)\left(f_{pot\_direct} cm_{pot\_direct\_ext}^{non-org} + \left(1 - f_{pot\_direct}\right) cm_{pot\_up\_ext}^{non-org}\right) \end{aligned}$$

- Rape seed is represented by “Raps, Grosshandel”. For the non-organic production system two intensity levels are distinguished (with f_{rape_int} being the fraction of intensive production). Accordingly, the contribution margin cm_{rape} is calculated as follows:

$$cm_{rape} = f_{org\_rape}\, cm_{rape}^{org} + \left(1 - f_{org\_rape}\right)\left(f_{rape\_\mathrm{int}} cm_{rape\_\mathrm{int}}^{non-org} + \left(1 - f_{rape\_\mathrm{int}}\right) cm_{rape\_ext}^{non-org}\right)$$

- Sugar beet is represented by “Zuckerrüben, Grosshandel” and “Futterrüben”. For the non-organic production system two intensity levels are distinguished (with f_{sugar_int} being the fraction of intensive production). Accordingly, the contribution margin cm_{sugar} is calculated as follows (in analogy: fodder beet):

$$cm_{sugar} = f_{org\_sugar}\, cm_{sugar}^{org} + \left(1 - f_{org\_sugar}\right)\left(f_{sugar\_\mathrm{int}} cm_{sugar\_\mathrm{int}}^{non-org} + \left(1 - f_{sugar\_\mathrm{int}}\right) cm_{sugar\_ext}^{non-org}\right)$$

$$cm_{fodderbeet} = f_{org\_fodderbeet}\, cm_{fodderbeet}^{org} + \left(1 - f_{org\_fodderbeet}\right)\begin{pmatrix} f_{fodderbeet\_\mathrm{int}} cm_{fodeerbeet\_\mathrm{int}}^{non-org} \\ + \left(1 - f_{fodderbeet\_\mathrm{int}}\right) cm_{fodderbeet\_ext}^{non-org} \end{pmatrix}$$

Supplementary material to Schuwirth, N., Honti, M., Logar, I., Stamm, Ch.: Multi-criteria decision analysis for integrated water quality assessment and management support. *Water Research X*, doi:10.1016/j.wroa.2018.100010

- Other arable crops

- Beans, peas and soy: we distinguish between organic and non-organic farming and two intensity levels for the non-organic version. Accordingly, the contribution margins are calculated as follows:

$$cm_{beans} = f_{org\_beans}\, cm_{beans}^{org} + \left(1 - f_{org\_beans}\right)\left(f_{beans\_\mathrm{int}} cm_{beans\_\mathrm{int}}^{non-org} + \left(1 - f_{beans\_\mathrm{int}}\right) cm_{beans\_ext}^{non-org}\right)$$

$$cm_{peas} = f_{org\_peas}\, cm_{peas}^{org} + \left(1 - f_{org\_peas}\right)\left(f_{peas\_\mathrm{int}} cm_{peas\_\mathrm{int}}^{non-org} + \left(1 - f_{peas\_\mathrm{int}}\right) cm_{peas\_ext}^{non-org}\right)$$

$$cm_{soy} = f_{org\_soy}\, cm_{soy}^{org} + \left(1 - f_{org\_soy}\right)\left(f_{soy\_\mathrm{int}} cm_{soy\_\mathrm{int}}^{non-org} + \left(1 - f_{soy\_\mathrm{int}}\right) cm_{soy\_ext}^{non-org}\right)$$

3.5. Grassland

- Permanent grassland, intensive is represented by “Naturwiese intensiv”. For the non-organic production system two intensity levels are distinguished (with $f_{grass\_very-\mathrm{int}}$ being the fraction of very intensive production). Accordingly, the contribution margin cm_{grass_int} is calculated as follows:

$$cm_{grass\_\mathrm{int}} = f_{org}\, cm_{\mathrm{int}}^{org} + \left(1 - f_{org}\right)\left(f_{grass\_very-\mathrm{int}} cm_{grass\_very-\mathrm{int}}^{non-org} + \left(1 - f_{grass\_very-\mathrm{int}}\right) cm_{grass\_\mathrm{int}}^{non-org}\right)$$

- Permanent grassland, extensive is represented by “Naturwiese extensiv” with a distinction between organic and non-organic farming. In this case, the non-organic farming production has only one intensity level. Accordingly, the contribution margin cm_{grass_ext} is calculated as follows:

$$cm_{grass\_ext} = f_{org}\, cm_{grass\_ext}^{org} + \left(1 - f_{org}\right) cm_{grass\_ext}^{non-org}$$

- Rotational grassland is represented by “Kunstwiese”. For the non-organic production system two intensity levels are distinguished (with $f_{rot-grass\_\mathrm{int}}$ being the fraction of intensive production). Accordingly, the contribution margin $cm_{rot-grass}$ is calculated as follows:

$$cm_{rot-grass} = f_{org\_rot-grass}\, cm_{rot-grass}^{org} + \left(1 - f_{org\_rot-grass}\right)\left(f_{rot-grass\_\mathrm{int}} cm_{rot-grass\_\mathrm{int}}^{non-org} + \left(1 - f_{rot-grass\_\mathrm{int}}\right) cm_{rot-grass\_ext}^{non-org}\right)$$

3.6. Fruits

- Apples are represented by “Tafeläpfel”. For the non-organic production system one distinguishes between a production with and one without hail protection (with $f_{apple\_hail\_pro}$ being the fraction with hail protection). Accordingly, the contribution margin cm_{apple} is calculated as follows:

$$cm_{apple} = f_{org}\, cm_{apple}^{org} + \left(1 - f_{org}\right)\left(f_{apple\_with} cm_{apple\_with}^{non-org} + \left(1 - f_{apple\_with}\right) cm_{apple\_non}^{non-org}\right)$$

- Grapes: We consider “Blauburgunder, ZH”. The contribution margin is given by:

$$cm_{grapes} = f_{org}\, cm_{grapes}^{org} + \left(1 - f_{org}\right) cm_{grapes}^{non-org}$$

Supplementary material to Schuwirth, N., Honti, M., Logar, I., Stamm, Ch.: Multi-criteria decision analysis for integrated water quality assessment and management support. *Water Research X*, doi:10.1016/j.wroa.2018.100010

3.7. Vegetables

For vegetables, there are no specific data for our catchment available. For simplicity, we have selected carrots as the most wide-spread crop in this category as a placeholder (Möhring et al., 2012). We simply distinguish between organic and non-organic production. Accordingly, the contribution margin $cm_{vegetables}$ is calculated as follows:

$$cm_{vegetables} = f_{org}\, cm_{carrots}^{org} + \left(1 - f_{org}\right) cm_{carrots}^{non-org}$$

3.8. Animal production

Dairy cows: we assume that all farms are in the silage area (no production of hard cheese). For organic and non-organic farms we distinguish between three levels of milk production per cow and year (5000, 6000 and 7000kg for organic, 7000, 8000 and 9000kg for non-organic farms). The corresponding fractions are given by $f_{dairy\_5000}^{org}$, $f_{dairy\_6000}^{org}$, $f_{dairy\_7000}^{non-org}$, and $f_{dairy\_8000}^{non-org}$. Accordingly, the contribution margin cm_{dairy} per cow equivalent is by:

$$\begin{aligned} cm_{dairy} = {} & f_{org}\begin{pmatrix} f_{dairy\_5000}^{org} cm_{dairy\_5000}^{org} + f_{dairy\_6000}^{org} cm_{dairy\_6000}^{org} \\ + \left(1 - f_{dairy\_5000}^{org} - f_{dairy\_6000}^{org}\right) cm_{dairy\_7000}^{org} \end{pmatrix} \\ & + \left(1 - f_{org}\right)\begin{pmatrix} f_{dairy\_7000}^{non-org} cm_{dairy\_7000}^{non-org} + f_{dairy\_8000}^{non-org} cm_{dairy\_8000}^{non-org} \\ + \left(1 - f_{dairy\_7000}^{non-org} - f_{dairy\_8000}^{non-org}\right) cm_{dairy\_9000}^{non-org} \end{pmatrix} \end{aligned}$$

Beef: for non-organic farms we consider the categories “Zuchtrind” and “Mutterkuh” with $f_{beef\_cow}^{non-org}$ giving the fraction of “Mutterkuh”; for organic farms there is only the category “Mutterkuh”. For each of these categories there are three sub-classes (“Zuchtrind”: “own breed”, “first calf with 26 months” and “first calf with 32 months”; “Mutterkuh”, organic: “Natura-Beef”, “Remont” and “Pasture”; “Mutterkuh”, non-organic: “SwissPrimBeaf”, “Natura-Beef” and “Natura-Veal”).

The respective fractions are labeled as follows: “Mutterkuh”, organic: $f_{beef\_Natura-Beef}^{org}$, $f_{beef\_Remont}^{org}$; “Mutterkuh”, non-organic: $f_{beef\_SwissPrim}^{non-org}$, $f_{beef\_Natura-Beef}^{non-org}$ and “Zuchtrind” with $f_{beef\_own}^{non-org}$, $f_{beef\_26mo}^{non-org}$. Accordingly, the contribution margin cm_{beef} per cow equivalent is by:

$$\begin{aligned} cm_{beef} = {} & f_{org}\begin{pmatrix} f_{beef\_Natura-Beef}^{org} cm_{beef\_Natura-Beef}^{org} + f_{beef\_Remont}^{org} cm_{beef\_Remont}^{org} \\ + \left(1 - f_{beef\_Natura-Beef}^{org} - f_{beef\_Remont}^{org}\right) cm_{beef\_past}^{org} \end{pmatrix} \\ & + \left(1 - f_{org}\right) f_{beef\_cow}^{non-org}\begin{pmatrix} f_{beef\_SwissPrim}^{non-org} cm_{beef\_SwissPrim}^{non-org} + f_{beef\_Natura-Beef}^{non-org} cm_{beef\_Natura-Beef}^{non-org} \\ + \left(1 - f_{beef\_SwissPrim}^{non-org} - f_{beef\_Natura-Beef}^{non-org}\right) cm_{beef-Veal}^{non-org} \end{pmatrix} \\ & + \left(1 - f_{org}\right)\left(1 - f_{beef\_cow}^{non-org}\right)\begin{pmatrix} f_{beef\_own}^{non-org} cm_{beef\_own}^{non-org} + f_{beef\_26mo}^{non-org} cm_{beef\_26mo}^{non-org} \\ + \left(1 - f_{beef\_own}^{non-org} - f_{beef\_26mo}^{non-org}\right) cm_{beef-32mo}^{non-org} \end{pmatrix} \end{aligned}$$

Pigs: we only consider the category “Mastschwein” with three feeding regimes (“corn-cob-mix (CCM)”, “whey (Schotte)”, “complete feedingstuff”) for the non-organic farms (fractions given by: $f_{pig\_CCM}^{non-org}$, $f_{pig\_Schotte}^{non-org}$) and two regimes (“complete feedingstuff” (= 700g daily weight gain), “whey” (= 650g daily weight gain) for organic farms ($f_{pig\_700g}^{org}$). Accordingly, the contribution margin cm_{pig} per pig (1/6 of a dairy cow equivalent) is given by:

$$cm_{pig} = f_{org}\left(f_{pig\_700g}^{org} cm_{pig\_700g}^{org} + \left(1 - f_{pig\_700g}^{org}\right) cm_{pig\_650g}^{org}\right)$$
$$+\left(1 - f_{org}\right)\left(f_{pig\_CCM}^{non-org} cm_{pig\_CCM}^{non-org} + f_{pig\_Schotte}^{non-org} cm_{pig\_Schotte}^{non-org} + \left(1 - f_{pig\_CCM}^{non-org} - f_{pig\_Schotte}^{non-org}\right) cm_{pig\_compl}^{non-org}\right)$$

Sheep: We consider meat and wool production and organic versus non-organic farming.

$$cm_{sheep} = f_{org}\, cm_{sheep}^{org} + \left(1 - f_{org}\right) cm_{sheep}^{non-org}$$

3.9. Set-aside areas

- The contribution margins of pasture (“Naturwiese, mittel-intensiv”), fallow (“Buntbrache auf Ackerland”) and mulch (“Streuwiese” $\cong$ ”Naturwiese, extensiv”) are calculated as e.g. for grapes. Both, organic and non-organic, production systems are considered, a further distinction of intensity types is not needed.

- The contribution margin of riparian hedges can directly be taken from the data. We do not distinguish organic/non-organic or intensive/extensive production systems.

References:

AGRIDEA, and FiBL: Deckungsbeiträge, Ausgabe 2012, Lindau, 48 S., 2012.

AWEL: Wasserqualität der Seen, Fliessgewässer und des Grundwasser im Kanton Zürich, Statusbericht 2006, Zürich, 2006.

Langhans, S. D., Lienert, J., Schuwirth, N., and Reichert, P.: How to make river assessments comparable: A demonstration for hydromorphology, Ecol. Indic., 32, 264-275, http://dx.doi.org/10.1016/j.ecolind.2013.03.027, 2013.

Liechti, P.: Methoden zur Untersuchung und Beurteilung der Fliessgewässer. Chemisch-physikalische Erhebungen, Nährstoffe. Umwelt-Vollzug Nr. 1005. Bundesamt für Umwelt, Bern. 44 S. , 2010.

Möhring, A., Mack, G., and Willersinn, C.: Gemüsebau - Modellierung der Heterogeneität und Intensität, Agrarforschung Schweiz, 3, 382 - 389, 2012.

4. Uncertainty estimation for costs

All parameters described in sections 2 and 3 were assumed to be uncertain. We performed a Monte-Carlo Simulation with a sample size of 3000 to propagate this uncertainty to the cost calculations to estimate the uncertainty of the costs of the management alternatives. The parameter distributions for all parameters are given in Table S 14 and Table S 15. In the absence of more detailed information, we chose normal distributions for parameters that are unconstrained, that can be both negative or positive, and where we had no indication of skewness. We chose lognormal distributions for parameters that cannot be negative. We chose truncated normal or lognormal distributions for parameters that can only take values between 0 and 1, depending on whether they should be symmetric or skewed.

Table S 14: Parameter distributions for urban management alternatives

Parameter name	Distribution	mean	sdev	min	max
pav_A_cost	Lognormal	42	5		
pav_B_cost	Lognormal	75	9		
pav_C_cost	Lognormal	95	7.5		
pav_D_cost	Lognormal	95	2.5		
pav_A_life	Lognormal	15	2.5		
pav_B_life	Lognormal	20	2.5		
pav_C_life	Lognormal	20	2.5		
pav_D_life	Lognormal	20	2.5		
inf_cost	Lognormal	26	20		
inf_life	Lognormal	25	2.5		
fac_cost_with	Lognormal	29.5	4		
fac_cost_without	Lognormal	32	4		
fac_life_with	Lognormal	20	5		
fac_life_without	Lognormal	15	5		
cso_const_a	Normal	82751	39470		
cso_const_b	Normal	-0.539	0.093		
cso_const_s	Normal	0	571		
cso_equip_a	Normal	3541	3214		
cso_equip_b	Normal	-0.389	0.171		
cso_equip_s	Normal	0	126		
cso_life_concr	Lognormal	40	5		
cso_life_equip	Lognormal	17.5	1.25		
wwtp_invest_a	Normal	34743	9133		
wwtp_invest_b	Normal	-0.549	0.029		
wwtp_invest_s	Normal	0	9		
wwtp_operation_a	Normal	79.4	46.6		
wwtp_operation_b	Normal	-0.261	0.06		
wwtp_operation_s	Normal	0	1		
wwtp_f_constr	NormalTrunc	0.5	0.1	0	1
wwtp_life_constr	Lognormal	30	4		
wwtp_life_equip	Lognormal	12	1.25		

Supplementary material to Schuwirth, N., Honti, M., Logar, I., Stamm, Ch.: Multi-criteria decision analysis for integrated water quality assessment and management support. *Water Research X*, doi:10.1016/j.wroa.2018.100010

Table S 15: Parameter distributions for urban management alternatives

Parameter name	Distribution	mean	sdev	min	max
forg	LognormalTrunc	0.2	0.15	0	1
fwheat.bread.int	LognormalTrunc	0.7	0.1	0	1
fdinkel.int	LognormalTrunc	0.01	0.1	0	1
ftri.int	LognormalTrunc	0.05	0.1	0	1
fbarley.int	LognormalTrunc	0.5	0.15	0	1
fcorn.silage.int	LognormalTrunc	0.99	0.1	0	1
fcorn.grain.int	LognormalTrunc	0.99	0.1	0	1
fpot.int	LognormalTrunc	0.99	0.1	0	1
fpot.dir	LognormalTrunc	0.9	0.25	0	1
fRape.int	LognormalTrunc	0.9	0.1	0	1
fSugar.int	LognormalTrunc	0.99	0.1	0	1
fFeedB.int	LognormalTrunc	0.99	0.1	0	1
fSoy.int	LognormalTrunc	0.99	0.1	0	1
fBeans.int	LognormalTrunc	0.99	0.1	0	1
fPeas.int	LognormalTrunc	0.99	0.1	0	1
fApp.with	LognormalTrunc	0.55	0.2	0	1
fIntGrass.int	LognormalTrunc	0.9	0.25	0	1
fRotGrass.int	LognormalTrunc	0.99	0.1	0	1
fbeef26	LognormalTrunc	0.35	0.2	0	1
fbeef32	LognormalTrunc	0.35	0.2	0	1
fbeefPrim	LognormalTrunc	0.35	0.2	0	1
fbeefVeal	LognormalTrunc	0.35	0.2	0	1
fnonorgsuckler	LognormalTrunc	0.35	0.2	0	1
fbeefBioNatura	LognormalTrunc	0.55	0.2	0	1
fbeefRemont	LognormalTrunc	0.35	0.2	0	1
fnonorg7000	LognormalTrunc	0.3	0.2	0	1
fnonorg8000	LognormalTrunc	0.55	0.2	0	1
forg5000	LognormalTrunc	0.3	0.2	0	1
forg6000	LognormalTrunc	0.55	0.2	0	1
fnonorgSchotte	LognormalTrunc	0.3	0.2	0	1
fnonorgCCM	LognormalTrunc	0.55	0.2	0	1
forgSchotte	LognormalTrunc	0.55	0.2	0	1
fwheat.feed.int	LognormalTrunc	0.01	0.1	0	1
forg.wheat.feed	LognormalTrunc	0.01	0.1	0	1
forg.wheat.bread	LognormalTrunc	0.09	0.15	0	1
forg.dinkel	LognormalTrunc	0.65	0.15	0	1
forg.tri	LognormalTrunc	0.09	0.15	0	1
forg.barley	LognormalTrunc	0.07	0.15	0	1
forg.corn.silage	LognormalTrunc	0.12	0.1	0	1
forg.corn.grain	LognormalTrunc	0.1	0.1	0	1
forg.pot	LognormalTrunc	0.32	0.1	0	1
forg.Rape	LognormalTrunc	0.01	0.1	0	1
forg.Sugar	LognormalTrunc	0.01	0.1	0	1
forg.FeedB	LognormalTrunc	0.01	0.1	0	1
forg.Soy	LognormalTrunc	0.01	0.1	0	1
forg.Beans	LognormalTrunc	0.45	0.1	0	1
forg.Peas	LognormalTrunc	0.2	0.1	0	1

forg.RotGrass	LognormalTrunc	0.24	0.1	0	1

5. Absolute costs for management alternatives

Table S 16: Absolute additional costs of management alternatives in CHF/year, exchange rate between Euros and Swiss Franks 1 EUR = CHF 1.1451 (European Central Bank 18.10.2018)

	BanBioc	StoreVol	PermPav	RainRet	WWTP	OrgFarm	NatPark	BufZone
StatusQuo	960000	395000	2850000	212000	606000	2990000	8220000	51200
Modgro	1110000	395000	2990000	222000	671000	2970000	8140000	50700
Expgro	6410000	395000	382000	827000	2050000	1330000	3660000	22800
Decline	960000	395000	2850000	212000	606000	2990000	8220000	51200

Supplementary material to Schuwirth, N., Honti, M., Logar, I., Stamm, Ch.: Multi-criteria decision analysis for integrated water quality assessment and management support. *Water Research X*, doi:10.1016/j.wroa.2018.100010

6. R-Script Example Implementation of Value functions

```
# R-script example for the definition of a value function ####
#         for water quality assessment
# by Nele Schuwirth, nele.schuwirth@eawag.ch
# 23.10.2018
# .......................................... ................

# package installation / loading ####

if ( !require(utility) )
{
  install.packages("utility")
  library(utility)
}

# definition of endnodes  ####

c_eqs_A <- 0.5
c_eqs_B <- 1
c_eqs_C <- 2

c_worst_A <- 5
c_worst_B <- c_eqs_B*3
c_worst_C <- c_eqs_C*40

c_best <- 0

substanceA <-  # two classes (passed/failed)
  utility.endnode.intpol1d.create(
    name.node   = "substance A",
    name.attrib = "conc. A mgperL",
    range       = c(c_best,c_worst_A),
    x           = c(c_best,c_eqs_A,c_worst_A),
    u           = c(1,0.6,0),
    required    = FALSE,
    utility     = FALSE)

substanceB <- # 5 classes, class boundaries according to Swiss nutrient assessment
  utility.endnode.intpol1d.create(
    name.node   = "substance B",
    name.attrib = "conc. B ugperL",
    range       = c(c_best,c_worst_B),
    x           = c(c_best,0.5*c_eqs_B,c_eqs_B,1.5*c_eqs_B,2*c_eqs_B,c_worst_B),
    u           = c(1,0.8,0.6,0.4,0.2,0),
    required    = FALSE,
    utility     = FALSE)

substanceC <- # 5 classes, class boundaries according to upcoming Swiss micropoll assessment
  utility.endnode.intpol1d.create(
    name.node   = "substance C",
    name.attrib = "conc. C mgperL",
    range       = c(c_best,c_worst_C),
    x           = c(c_best,0.1*c_eqs_C,c_eqs_C,2*c_eqs_C,10*c_eqs_C,c_worst_C),
    u           = c(1,0.8,0.6,0.4,0.2,0),
    required    = FALSE,
    utility     = FALSE)

# plot of endnodes ####

plot(substanceA,col = c("red","red","red","green","green"))
plot(substanceB)
plot(substanceC)

# definition aggregation node ####
# with additive minimum aggregation

chemicalstate <-
  utility.aggregation.create(
```

```
    name.node    = "chemical state",
    nodes        = list(substanceA,substanceB,substanceC) ,
    name.fun     = "utility.aggregate.addmin",
    par          = c(1,1,1,0.5),
    names.par    = c("w_A","w_B","w_C","w_add"),
    required     = FALSE,
    num.required = 3,
    col          = "black",
    shift.levels = 0,
    add.arg.fun  = NULL)

# plot aggregation node ####
plot(chemicalstate)

# define attribute values for 4 hypothetical alternatives ####

attrib <- as.data.frame(matrix(nrow=4,ncol=3))
rownames(attrib) <- c("Alternative 1","Alternative 2","Alternative 3","Alternative 4")
colnames(attrib) <- c("conc. A mgperL","conc. B ugperL","conc. C mgperL")
attrib[,"conc. A mgperL"] <- c(1,0.5,0.9,0.01)
attrib[,"conc. B ugperL"] <- c(0,3,1.5,0.1)
attrib[,"conc. C mgperL"] <- c(40,1,0.02,3)

# calculate results ####

res.chem <- evaluate(chemicalstate,attrib)

# plot results ####

par(mar=c(0,0,0,0))
# plot color-coded hierarchy for Alternative 1
plot(chemicalstate,u=res.chem[1,],type="hierarchy",main=rownames(res.chem[1]))

# plot color-coded table for all 4 alternatives
plot(chemicalstate,u=res.chem,type="table")
```

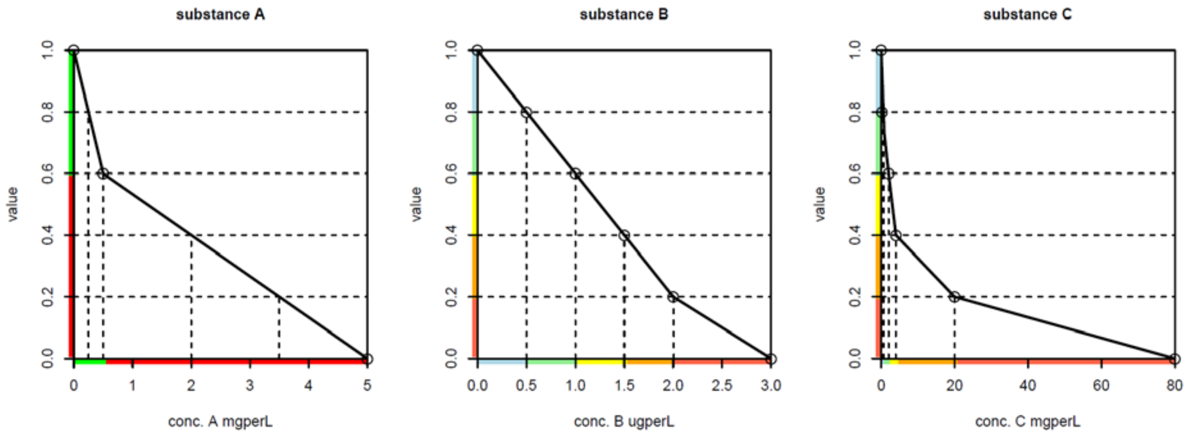

Fig. S 6: Plot of endnodes, i.e. hypothetical value functions.

Supplementary material to Schuwirth, N., Honti, M., Logar, I., Stamm, Ch.: Multi-criteria decision analysis for integrated water quality assessment and management support. *Water Research X*, doi:10.1016/j.wroa.2018.100010

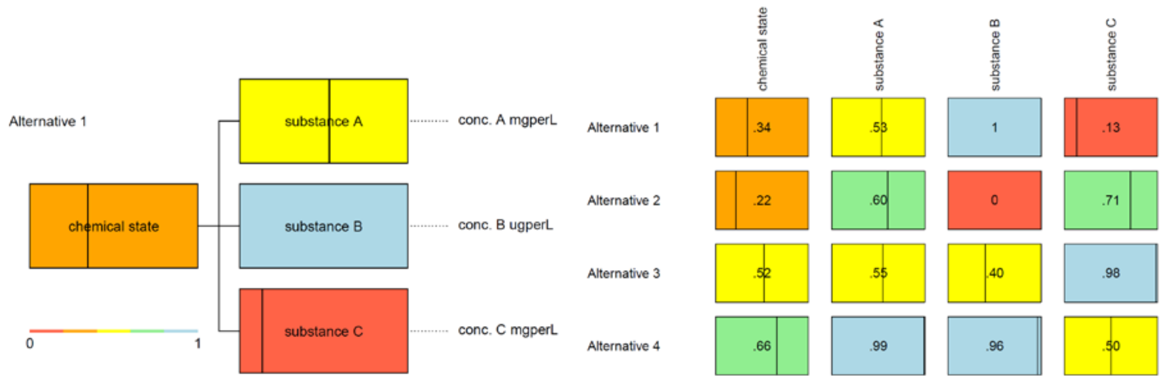

Fig. S 7: Plot color-coded hierarchy for Alternative 1 (left panel) and color-coded table for all hypothetical alternatives (right panel).17

Supplementary material to Schuwirth, N., Honti, M., Logar, I., Stamm, Ch.: Multi-criteria decision analysis for integrated water quality assessment and management support. *Water Research X*, doi:10.1016/j.wroa.2018.100010
